# Human influence on Amazon’s aboveground carbon dynamics intensified over the last decade

**DOI:** 10.1038/s41467-025-61856-1

**Published:** 2025-07-21

**Authors:** Arthur Fendrich, Yu Feng, Jean-Pierre Wigneron, Jerôme Chave, Arnan Araza, Zheyuan Li, Martin Herold, Jean Ometto, Luiz E. O. C. Aragão, Isabel Martinez Cano, Lei Zhu, Yidi Xu, Philippe Ciais

**Affiliations:** 1 Laboratoire des Sciences du Climat et de l’Environnement, UMR 1572 CEA-CNRS-UVSQ, Gif-sur-Yvette, France; 2 European Commission, Joint Research Centre (JRC), Ispra, Italy; 3 Eastern Institute for Advanced Study, Eastern Institute of Technology, Ningbo, China; 4 INRAE, Bordeaux Sciences Agro, UMR 1391 ISPA, Villenave-d’Ornon, France; 5 Centre de Recherche Biodiversite Environnement, CNRS, IRD, UPS, INPT, Toulouse, France; 6 Earth Systems and Global Change, Wageningen University and Research, Wageningen, The Netherlands; 7 School of Mathematics and Statistics, Henan University, Kaifeng, China; 8 Institute for Environmental Science and Geography, University of Potsdam, Potsdam, Germany; 9 Remote Sensing and Geoinformatics Section, Helmholtz GFZ German Research Centre for Geosciences, Telegrafenberg, Potsdam, Germany; 10 Impact, Adaptation and Vulnerability Division, Instituto Nacional de Pesquisas Espaciais, São José dos Campos, Brazil; 11 Earth Observation and Geoinformatics Division, Instituto Nacional de Pesquisas Espaciais, São José dos Campos, Brazil; 12 Geography, University of Exeter, Exeter, EX4 4PY UK; 13 Department of Earth System Science, Ministry of Education Key Laboratory for Earth System Modeling, Institute for Global Change Studies, Tsinghua University, Beijing, China

**Keywords:** Climate and Earth system modelling, Carbon cycle

## Abstract

The Amazon rainforest is crucial for the global carbon cycle, yet annual changes in its aboveground biomass carbon (AGC) stock remain highly uncertain. Natural and local anthropogenic drivers such as deforestation, forest degradation, and regrowth following deforestation interact with large-scale climate variability to determine AGC dynamics. Here, we propose an approach to disaggregate low-frequency passive L-band microwave data over 2010-2020 and reconstruct maps of annual change. We show that the Amazon lost −0.37 ± 0.17 PgC, with gains by undisturbed (0.33 ± 0.13 PgC) and secondary forest growth (0.33 ± 0.05 PgC) outweighed by losses by deforestation (−0.55 ± 0.04 PgC), degradation (−0.42 ± 0.08 PgC), and agricultural areas (−0.06 ± 0.03 PgC). Losses in human-influenced land intensified over time and amounted to 60% of all gross losses in El Niño years. Our study reinforces the need for stronger implementation of policies and effective actions to control forest degradation.

## Abstract (web summary)

New high-resolution datasets for the Amazon forest show a loss of carbon in 2010-2020, with gains by forest growth outweighed by losses by deforestation and degradation. Human losses intensified over time, reinforcing the need for stronger policies.

## Introduction

The Amazon rainforest plays a central role in the global carbon cycle. Accounting for 14% of the total atmospheric carbon fixed by plants annually^1^, it stores 102 ± 26 PgC in aboveground biomass carbon (AGC)^2^, equivalent to 28% of global AGC^3,4^. Human activity has reduced the carbon storage capacity of the Amazon through deforestation and forest degradation, with 20% of its original extent converted to other land uses and 5.5% of the remaining forests degraded by fire, logging and fragmentation^5,6^. Yet, despite a reduction in deforestation levels since 2005, the Amazon has been cleared at an average rate of 7927 km^2^ year^−1^ since 2010, with an increasing trend since 2016, and degradation continues to increase^7^. Recent vertical profiling measurements of atmospheric CO_2_ concentration showed that the southeastern part of the forest has become a net carbon source^8^, pointing toward a decline of the Amazon forest sink of 0.37 PgC year^−1^ between 2001 and 2019^9,10^. The effective implementation of policies for forest conservation requires an improved understanding of the dynamics of all components and processes that lead to biomass change, and an attribution if such changes happen in natural or human-influenced land.

The Vegetation Optical Depth derived from L-band passive microwave data of the Soil Moisture and Ocean Salinity (SMOS) satellite (hereinafter referred to as L-VOD) has been used to map AGC changes at annual timescales^11^. L-VOD measures the extinction effects of the vegetation affecting the microwave propagation within the canopy layer and quantifies vegetation water content in the photosynthetic and woody components of the plants^12^. L-VOD appears to be a better proxy for AGC than vegetation indices derived from optical sensors due to its greater penetration capacity in woody canopies and lower sensitivity to saturation effects in dense forests^13^. Trends in L-VOD are little sensitive to the effects of the moisture content of vegetation and the temporal variations are mostly driven by biomass changes^14^. Moreover, L-VOD is unaffected by clouds, a particularly desirable property for tropical rainforests^15^. The major drawback of L-VOD, however, is its coarse spatial resolution of about 25 km at the Equator^12^. Therefore, if L-VOD represents well the net dynamics of biomass carbon change, it averages out changes due to local sub-grid processes such as the losses from deforestation or degradation and the gains from forest growth or regrowth at finer scales^16^. Efforts to overcome this limitation include the European Space Agency Climate Change Initiative (CCI) Biomass project^17,18^, which combined the C-band of ENVISAT ASAR with ALOS-1 Phased Array L-band SAR (PALSAR-1) and the active synthetic aperture radar C-band of Sentinel-1 with the L-band of ALOS-2 PALSAR-2 to generate annual AGC maps for the period 2010, and 2017–2020 at a 100-m spatial resolution, in its fourth version. Although the dataset provides high-resolution estimates of AGC, temporal gaps and biases between years due to the use of different satellite sensors for different epochs limit its performance over the Amazon, and preclude its use for consistent multi-year change detection^17,18^. At the Jet Propulsion Laboratory, Xu et al.^19^ derived a temporally consistent AGC dataset through the period spanning 2000–2019 (hereinafter referred to as JPL) from a combination of optical and microwave satellite data, but the spatial resolution of around 10 km at the Equator hinders the understanding of fine-scale processes. Furthermore, the modified carbon-flux model of Araza et al.^20,21^ (hereinafter referred to as WRI) estimates AGC changes in the period 2010–2019 at a 100-m spatial resolution by combining the baseline CCI map for 2010 with annual conversion factors.

Despite the aforementioned efforts, most of the bias in existing global AGC maps appears in tropical forests, and the Amazonian region has significant disagreements in terms of AGC change through time^20–22^. Therefore, a potential pathway to improve the existing knowledge is the development and application of reliable methods to downscale L-VOD information from its original coarse resolution to a finer scale^23^, because AGC changes are inferred from consistent repeated measurements over the entire period. The high-resolution maps generated from such an effort would open up the possibility of improving the reporting of national greenhouse gas emissions in biennial transparency reports, advancing the modeling representation of carbon turnover or its allocation to plant tissue^24^, and refining spatiotemporal patterns of AGC change^25^.

Here, we aim at dividing the AGC changes in the Amazon rainforest into those that have occurred in natural or human-influenced land (see “Materials and Methods” for details). We applied a constrained statistical disaggregation model to L-VOD time series to generate a time series of annual sub-grid AGC changes in Amazonian forests from 2010 to 2020 at 100-m spatial resolution, with results that converge to the coarse resolution L-VOD AGC estimates. As inputs to the disaggregation model, we incorporated reference datasets for dynamic factors that affect the AGC change, including human-induced activity changes (degradation, deforestation, and regrowth^26^), relevant climate variables (air temperature and water deficit), as well as time-varying curves representing the recovery from degradation and the growth of old and new forests^27^. The new maps of annual AGC changes during 2010-2020 generated at the high resolution of 100 m were used to calculate the gross and net carbon budget, and the dynamics of natural and human-influenced land uses through time since 2010.

## Results and discussion

### Reconstructed patterns

**Fig. 1:**
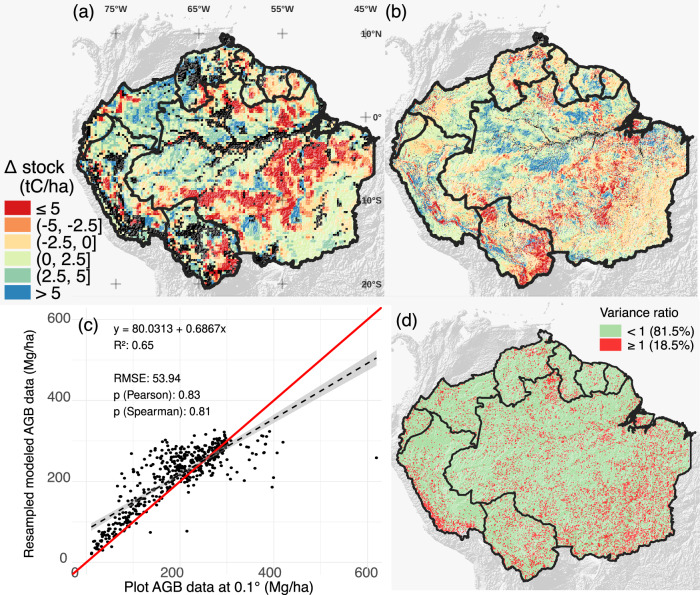
Spatial pattern of aboveground biomass carbon (AGC) changes the Amazon biome. **a**–**d**: (**a**) change of AGC 2010-2020 (∆) from vegetation optical depth (VOD) measurements at a coarse 0.25° spatial resolution (~ 27.8 km at the Equator), **b** reconstructed delta AGC 2010-2020 at high spatial resolution (~100-m at the Equator), (**c**) comparison of 100 m AGC stocks against forest plot data at a 0.1° spatial resolution, with a 1:1 line in red, and (**d**) the variance ratio of biomass change (2010-2018) with and without our reconstruction. Black pixels in (**a**, **b**) represent flooded areas and masked outliers (see “*Materials and Methods”*).

The spatial patterns of our reconstructed fine-scale AGC changes between 2010 and 2020 in the Amazon were compared against the original, coarse resolution AGC estimates (Fig. 1a, b), evaluated against forest plot data aggregated to a 0.1° spatial resolution (Fig. 1c), and compared with other AGC products (Fig. 1d). Supplementary sections C2 to C5 present further validations and comparisons, including a validation at high resolution against Light Detection and Ranging (LiDAR) data. The last comparison shows that CCI data has a higher linear correlation with LiDAR than our maps across all land cover types, but our maps often exhibit lower root mean square error than CCI (Table S1, Table S2). Our reconstructed high-resolution values of AGC change ranged between -204 and 371 MgC ha^−1^, with 99% of the changes between -64 and 34 MgC ha^−1^, and with a median of 0.2 MgC ha^−1^, respectively. The major losses occurred in areas of intense deforestation and degradation, such as the arc of deforestation in Brazil and the Santa Cruz department in Bolivia. The comparison of reconstructed AGC values with field estimates yielded a Pearson correlation coefficient of 0.83 and a root mean squared error of 53.94 MgC ha^−1^ (*n* = 482 cells containing forest plots, Fig. 1c, see Fig.S6 and Fig.S7 for comparison with other datasets). The analysis of convergence among datasets showed reduced uncertainty across 81.5% of the study domain (Fig. 1d). Such a reduction was characterized by a variance ratio comparing the uncertainty across our dataset combined with JPL^19^, CCI^17,18^ and WRI^20,21^, to the uncertainty among JPL, CCI and WRI alone. This finding indicates that our dataset contributes to consolidating the existing knowledge on the trends and spatial patterns of AGC changes in the Amazon. Our data-driven consistent time series of AGC change maps simultaneously agreed with existing sources at different spatial scales, opening new possibilities for spatial analyses in the Amazon.

The map of AGC stocks (Supplementary Material, Fig.S6) indicates a pattern of low biomass values in areas with intense direct human activity (e.g., eastern Brazil) or with a mountainous topography (e.g., western Peru), and higher AGC values when far from these areas. At the borders between undisturbed and non-undisturbed areas (Fig.S6), medium values may represent a loss of AGC possibly due to spillover effects of deforestation and degradation due to fragmentation, such as drier microclimate and other edge effects^28^. The 2020 map presents median AGC values of 132 MgC ha^−1^ in undisturbed and 69 MgC ha^−1^ in degraded forests (i.e., according to the TMF dataset^26^, see “Materials and Methods”). These values are lower than the 179 MgC ha^−1^ for undisturbed and similar to the range of 48-86 MgC ha^−1^ for degraded areas reported by forest inventory plots of ref. ^29^. AGC estimates in pixels with secondary forest regrowth in the Peruvian Amazon (Fig.S23) averaged 74 ± 35 MgC ha^−1^, higher than airborne LiDAR estimates on the Peruvian Amazon^30^ (i.e., 33 ± 7 MgC ha^−1^), and the same relationship held for deforested areas, at 38 ± 24 MgC ha^−1^ in our results versus 28 ± 17 MgC ha^−1^ in the LiDAR estimates^30^. In the Brazilian arc of deforestation, we estimated average gross losses of -97.8 TgC year^−1^ and gross gains of 37.8 TgC year^−1^ in 2016-2018, comparable to the −134.6 TgC year^−1^ and the 44.1 TgC year^−1^ estimated from LiDAR data by ref. ^31^. For the limitations of our deforestation and regrowth estimates, see sections C5 and C6 of the Supplementary Material, where the following subjects are evaluated: i) validation at finer scale; ii) distribution of disaggregated AGC stocks and limitations of L-VOD AGC; iii) effect of the land cover change dataset; iv) effect of the AGC input dataset; and v) impact of forcing model behavior.

### High-resolution AGC change

**Fig. 2:**
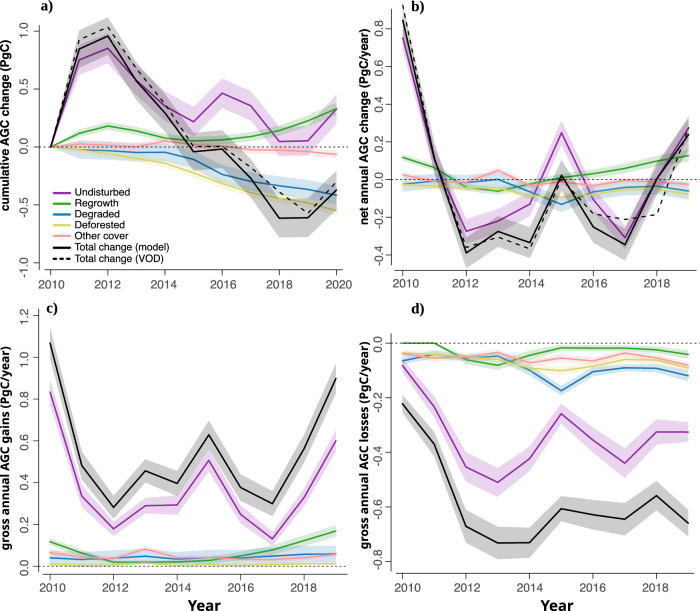
High-resolution reconstructed aboveground carbon (AGC) budget for the Amazon Biome over 2010-2020. **a**, **b** Cumulative AGC change split by land cover category, and comparison against raw VOD (**a**), net annual AGC change per land cover category (**b**), gross annual AGC gains per land cover category (**c**), and gross annual AGC losses per land cover category (**d**). The gross AGC change corresponds to the separation of losses and gains within a land cover category. For visualization purposes, uncertainty bands correspond to 1 times the standard deviation in (**a**) and 0.5 times the standard deviation in (**b**), (**c**) and (**d**). In the case of annual changes, values assigned to “year” were calculated as Stock[year+1] - Stock[year]. The “Total change (VOD)” line refers to the original VOD budget at a coarse resolution, while the “Total change (model)” line refers to the reconstructed high-resolution budget.

We analyzed the high-resolution net AGC change in the Amazonian rainforest with respect to the reference year of 2010, considering the contribution of each class: undisturbed forests, areas with regrowth, degraded forests, deforested areas, and other land cover types. Figure 2 shows the original coarse-scale budget derived from L-VOD directly with a dashed black line, and the reconstructed high-resolution budget with a continuous black line. The later information shows that the total net AGC change reached a maximum as stocks increased by 0.96 ± 0.16 PgC (mean ± standard deviation) over 2010–2012 and a minimum when they decreased by -0.61 ± 0.17 PgC over 2010-2018, respectively. Changes in stocks of undisturbed forests explain much of the annual variation in the first two years of the decade (Fig. 2b-d). These forests thrive in a dynamic equilibrium between biomass accumulation, continuous turnover, and discrete losses associated with natural disturbances such as droughts, insect outbreaks and pathogens, or windthrows^32^. After 2012, the increased participation of degradation and regrowth outweighed the contribution of undisturbed forests. The losses of AGC in undisturbed forests found in 2016 and 2017 matches the period when losses reached their highest levels in the region after the El Niño drought of 2016^25^. As a result, a net cumulative loss of -0.37 ± 0.17 PgC was calculated for 2010-2020, resulting from sinks of 0.33 ± 0.13 and 0.33 ± 0.05 PgC in undisturbed and secondary forests, respectively, offset by sources of -0.06 ± 0.03, -0.42 ± 0.08 and -0.55 ± 0.04 PgC by other land covers, degraded forests, and deforested areas, respectively (Table S3).

The results can be compared to those from the bottom-up carbon accounting model to disentangle AGC changes of Fawcett et al. ^11^. Within the same simulation domain (Fig.S23), the annual source due to deforestation and sink from regrowth (Fig.S25) are lower than those of Fawcett et al. ^11^. Degradation contributed to net emissions of −34 ± 29 TgC year^−1^ and deforestation amounted to −45 ± 18 TgC year^−1^, while Fawcett et al. ^11^ predicted around −37 ± 27 for degradation in non-edge areas, −54 ± 15 in edge areas and −135 ± 44 TgC year^−1^ for deforestation. Our deforestation estimates, however, do not depart much from -57.1 TgC year^−1^ obtained as the product between the average AGC in undisturbed forests (i.e., 134.86 MgC ha^−1^, Table S6), average deforestation rate (i.e., 14,595 km^2^ year^−1^, Table S6), and the fraction of biomass lost after fire events in the Amazon estimated by Anderson et al. (i.e., 29.16%)^33^. Secondary forests represented a sink of 20 ± 38 TgC year^−1^, much lower than the 42 ± 2 TgC year^−1^ found by ref. ^11^. Undisturbed forests were a net sink with an average of 26 ± 267 TgC year^−1^, which is comparable to the sink in old-growth forests of 36 ± 197 TgC year^−1^ in^11^. Although informative, such a comparison is not conclusive due to methodological differences between the two works. Such differences comprise the use of different datasets to map deforestation, degradation and regrowth, and the distinction between edge and non-edge degradation in ref. ^11^, which is absent in the current work.

Gross annual AGC gains were 0.55 ± 0.26 PgC year^−1^ over 2010–2020 (Fig. 2c). This gross gain occurred mainly in undisturbed forests, responsible for a gain of 0.37 ± 0.21 PgC year^−1^, while the contribution of secondary forests was only 0.07 ± 0.05 PgC year^−1^, similar to that from the recovery of degraded forests of 0.04 ± 0.01 PgC year^−1^. These values correspond to an average gross annual accumulation rate of 1.3 ± 0.3, 16.3 ± 7.3, and 1.6 ± 0.2 MgC ha^−1^ year^−1^ by undisturbed, secondary and recovering degraded forest, respectively (Table S3). Gross AGC gains in non-forest land cover types (i.e., agriculture, savanna, shrubland, and non-vegetated areas^26^) and in previously and newly deforested areas were very small, with 0.05 ± 0.02 and 0.01 ± 0.003 PgC, respectively. The gross annual AGC loss was found to be −0.58 ± 0.16 PgC year^−1^ (Table S3) over the same period, most of which came from natural forest areas (−0.34 ± 0.13 PgC year^−1^), degraded forests (-0.09 ± 0.04 PgC year^−1^) and deforestation (−0.07 ± 0.02 PgC year^−1^). Other land cover types, −0.05 ± 0.02 PgC year^−1^, and secondary forests, -0.03 ± 0.03 PgC year^−1^, presented smaller gross losses. Our results align with those of^27^, which described that although forest degradation leads to lower losses per unit area compared to deforestation, it leads to higher total emissions due to the larger area affected^6,27^. Besides, degradation losses per unit area can increase over time, since forest degradation events occur repeatedly and can lead to deforestation. We calculated a contribution of degradation to the annual gross losses of 16 ± 8% (Fig. 2b-d), below the 30% of^34^, the 33.3% of selective logging in ref. ^35^ for drought years, but within the 18-40% range reported in local inventories and bookkeeping models^27^. The 65.6% contribution of gross losses by degradation reported by ref. ^35^ for non-drought years and the 73% found by another model using VOD data^27^ are close to the 56 ± 6% that we find as the proportion of losses due to degradation relative to the sum of degradation plus deforestation (Fig.S24, and Table S3).

### Human influence and implications

**Fig. 3:**
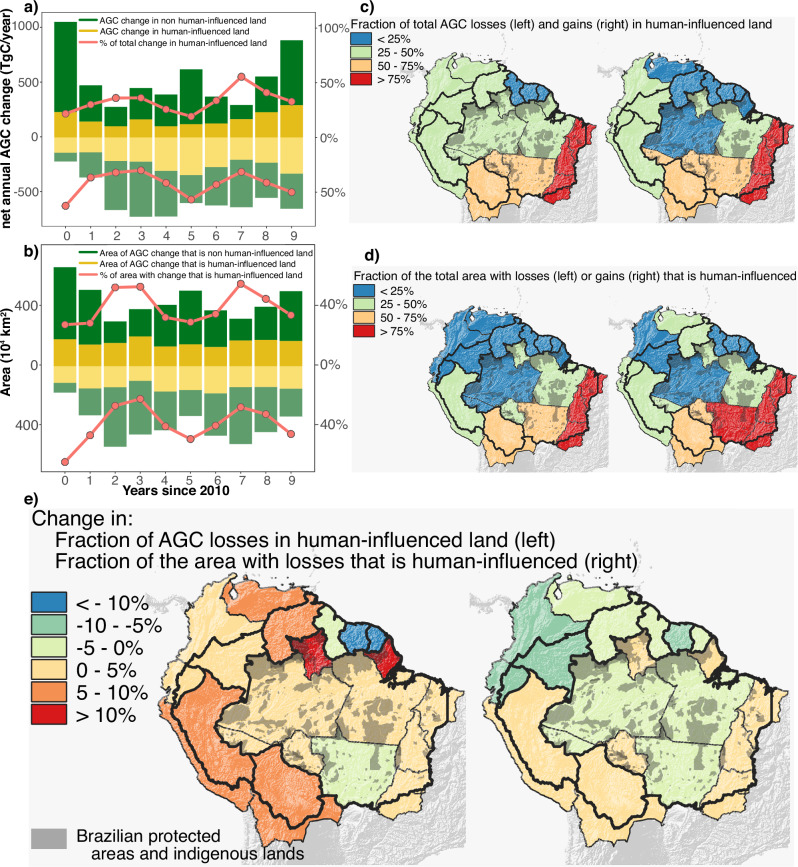
Annual changes in aboveground carbon (AGC) attributed to natural or human-influenced land and their corresponding spatial patterns. **a**–**d** Attribution of annual AGC change (**a**), attribution of area with AGC losses and gains (**b**); The share of total AGC losses [left] and gains [right] that happened in human-influenced land, averaged over 2015-2019 (**c**), and the share of the total area with losses [left] and gains [right] that were human-influenced land, averaged over 2015-2019 (**d**); Difference from the first to the second half of the decade in: the share of the total AGC losses that happened in human-influenced land [left], and the share of the total area with losses that were human-influenced land [right] (**e**). In the case of annual changes, values assigned to “year” were calculated as Stock[year+1] - Stock[year]. The “human-influenced” class corresponds to the sum of all classes except undisturbed forests.

Our attribution of AGC losses to natural or human-influenced land (see “Materials and Methods”) is presented in Fig. 3. Human-influenced lands were responsible for an annual gross loss of −242 ± 73 TgC year^−1^ and gain of 170 ± 65 TgC year^−1^, encompassing 43.0 ± 11.1% and to 33.5 ± 10.5% of the annual gross losses and gains, respectively (Fig. 3a). The share of losses in human-influenced land was exacerbated during El Niño episodes of 2010 and 2015^36,37^, as shown by the red line of losses in Fig. 3a. A high impact in human-influenced areas also happened in 2019-2020, the peak of deforestation during the study period^36^. In terms of area affected, human activities influenced 147 10^4 ^km² out of the total 330 10^4 ^km² (i.e., 44.5%) that had AGC losses in 2020, and 165 10^4 ^km² out of the total 493 10^4 ^km² (i.e., 33.5%) that had AGC gains in 2020 (Fig. 3b). The analysis per political region (i.e., federative unit in Brazil due to its larger size, and country elsewhere) provides meaningful insights. The fraction of the total area with gains that is human-influenced has a one-to-one correspondence with the fraction of the total AGC gains that they represent (Fig. 3c and d, right). However, for the losses, the proportionality deviates from a one-to-one relationship (Fig. 3c and d, left), indicating a variable intensity of forest degradation and deforestation in human-influenced land in the Amazon biome (Section C7.1, Table S7). The results show that the share of AGC losses in human-influenced land increased at a faster pace than the share of area of such activities (Fig. 3e, and Table S8), pointing to a pattern of significantly increased land use intensity from the first half (−1.57 MgC ha^−1^ year^−1^) to the second half of the last decade (−1.85 MgC ha^−1^ year^−1^, Section C7.2). Such a pattern, however, was not evenly distributed, with increases in Central and Northern regions and decreases in Eastern and Western regions (Fig.S29).

The analysis suggests a significant role of human activities in the AGC dynamics in the Amazon from 2010 to 2020, with changes in regional patterns. Inside Brazilian protected areas and indigenous lands (BPAs, Fig. 3e and Fig.S23), average gross losses by degradation increased from -7.8 to -17.1 TgC year^−1^ from the first to the second half of the decade, while losses by deforestation increased from -4.9 to -6.8 TgC year^−1^. Outside BPAs, average losses by degradation increased from -27.7 to -56.7 TgC year^−1^ in the same period, and losses by deforestation increased from -37.7 to -53.7 TgC year^−1^ (Table S4 and Table S5). In terms of intensity, losses in all human-influenced land inside BPAs increased from −2.0 to −2.8 MgC ha^−1^ year^−1^ ( + 40%), while outside BPAs the increase was less pronounced, from −1.4 to −1.6 MgC ha^−1^ year^−1^ ( + 13%). The intensification of AGC losses within BPAs is worrisome and raises concerns about the future of the Amazon rainforest. Sustained degradation and deforestation can accelerate regional climate change^38–42^, thus amplifying climate-related AGC losses. The implementation of policies aimed at reducing emissions due to deforestation and degradation will remain difficult if not based on near real-time and trusted maps, but the present study, together with other recent contributions, e.g.^43^, shows that this goal is within reach.

## Methods

### Input data and disaggregation model

The disaggregation model combines information from different spatial scales (see Supplementary section C1 and Fig.S22 for further details). First, we resampled L-VOD observations from 25 km to 0.25° (~27.8 km at the Equator, hereinafter referred to as the “coarse scale”). At such a coarse scale, we calibrated the relationships between L-VOD and benchmark AGC for the year 2011 following the methods of ref. ^44^, by fitting Eq. 1:

1 $${AGC}=d+a\frac{\arctan \left(b \, \left({VOD}-c\right)\right)-\arctan (-{b \, c})}{\pi /2-\arctan (-{b\,c})}$$

where *arctan* is the arc-tangent function, *AGC* is the aboveground biomass carbon [MgC ha^−1^], *VOD* is the L-VOD signal, and *a, b, c* and *d* [unitless] are the four parameters that were calibrated. We initially used L-VOD for 2011, and six AGC benchmark maps to generate the calibration functions with six sets of parameters (i.e., each AGC benchmark map with its own set of fitting parameters). The six AGC benchmark maps are the Avitabile map^45^, Baccini map^46^, CCI biomass 2010 map (version 4)^17,18^, two JPL maps^19,47,48^, and the Zarin map^49^. Then, we used these calibrations to derive six annual AGC map estimates for 2010-2020. The average of all estimates was used for the subsequent analysis. Details on the generation of annual L-VOD-based AGC maps can be found in ref. ^43^, ^50^. Since L-VOD observations have low quality in water bodies, we thus masked cell locations where more than 80% of the area regularly flooded and filtered out outliers (i.e., annual AGC gains of more than 20 MgC ha^−1^), following the approaches of^11^ and^37^. The masked areas can be seen in Fig. 1a.

The disaggregation approach then started with the definition of a statistical regression model (M1 of Fig.S22) at the fine scale (0.00089°, ~100 m at the Equator, hereinafter referred to as the “fine scale”), but since no field-level observations were available, the M1 could not be estimated. Next, to overcome this issue, we assumed that a coarse-resolution cell represents the average of all the fine-resolution AGC pixels within its spatial domain. We also assumed that fine-resolution pixels without any land cover assigned (e.g., water pixels) have a nil AGC and influence the average calculation at the coarse level. Then, since averaging is a linear operation, it can be shown that these assumptions lead to a second statistical model (M2 of Fig.S22) at the coarse level that depends on the original parameters of the high-resolution model^51^. Because the SMOS L-VOD data is available, M2 can be estimated, and its parameters can be plugged into M1 for prediction.

The linear predictors for the mean and variance terms then depend on explanatory variables at the fine scale. Among the potential variables, we considered the ESA CCI biomass map for the year 2010^17,18^, the high-resolution forest change (i.e., deforestation, degradation, and regrowth) from the European Commission’s Tropical Moist Forests (TMF)^26^ dataset at 30 m and annual intervals, and climatic variables time-series (i.e., maximum temperature and water deficit) at a 0.1° and a 0.05° spatial resolutions extracted from the ECMWF Reanalysis v5 (ERA5-Land)^52^ and the CHIRPS dataset^53^, respectively. The TMF dataset contains five classes: undisturbed, disturbed, deforestation, regrowth and other land cover. Given the evident dependence of the downscaling results on the TMF dataset used, more details about it can be found in Section C6 and Fig.S19. The boundaries of the Amazon basin were extracted from MapBiomas Amazonia^54^, and all the datasets were resampled and aligned to a 0.00089° resolution before processing, using either a bilinear (for climate variables) or nearest neighbor (for categorical variables) interpolation.

The generated outputs comprised the high-resolution maps of AGC for each year. An uncertainty layer containing an approximation to the lower bound of the standard deviation at the fine scale is also provided. To perform estimation at the fine scale, we allowed the extrapolation of the cubic splines on the domain covered by the coarse SMOS cells filtered out of the calibration procedure, and we recommend these extrapolated predictions to be used with extra care. For all analyses where uncertainty is reported, the solid lines correspond to the results obtained using the expected value of the estimated fine-resolution distribution, and the lower and upper bounds consist of a one standard deviation interval around the mean, unless stated otherwise.

### Fine-scale map assessment

Map assessment is defined as the comparison of the AGC map against reference data while accounting for the uncertainties of the latter, given that they are also not error-free^21^. Two analyses were made to perform such an assessment. First, our results were re-aggregated and compared to reference forest plot data at a 0.1° spatial resolution (~11.1 km at the Equator). The reference forest plot data was harmonized with our map and other AGC products of 2020 to address the temporal mismatch due to discrepancies between the year of plot surveys and map epochs, and the spatial mismatch due to differences in forest area definitions. Two steps were employed: (i) for temporal harmonization, AGC change in time was estimated through the use of auxiliary forest growth data; ii) for spatial harmonization, results of (i) were averaged at a 0.1° resolution and then multiplied by the corresponding forest cover share. A minimum of five plots per cell was defined to ensure the robustness of estimates, which led to an average of 11 plots per cell. All the procedures used are implemented in the open-source Plot2Map tool^55^, which was also used for the CCI Biomass independent validation framework^21^. Then, the map aggregation to 0.1° resolution was performed by calculating the weighted average of all high-resolution pixels. The weight assigned to each pixel was the inverse of its uncertainty (i.e., variance), and all land cover classes were included in the weighting procedure^21^. The forest biomass reference data used in this study came from different sources and consisted of collecting and systematically processing permanent research plots, national forest inventories, and local biomass maps derived from airborne LiDAR missions^29,42^. A detailed description of the reference data sources is provided in ref. ^21^. The accuracy indicators used to evaluate the maps included the Pearson and Spearman correlation coefficients and the root mean squared error (RMSE). Second, at a finer scale, model predictions were compared directly to the AGC data derived from the most extensive LiDAR campaign ever in the Brazilian Amazon to date, available at a 0.00225° spatial resolution (~250 m at the Equator)^42^. Such a dataset constitutes the most reliable large-scale information at a high resolution inside our study domain. Results are presented in the Supplementary Material (section C5).

### Uncertainty reduction analysis

To assess the contribution of our maps to the existing knowledge on AGC changes in the Amazon in the literature, we first gathered other publicly available datasets covering the same region and time period, namely JPL^19^, CCI^17^ and WRI^20,21^ at a 0.1°, 0.00089° and 0.00025° spatial resolution, respectively. These datasets and our work overlap from 2010 to 2018. Therefore, we calculated the difference of AGC from 2010 to 2018, adopting a factor of 50% to convert AGB to AGC when appropriate^25^. Then, we upscaled all datasets to the coarsest resolution among them (i.e., 0.1°) by averaging the pixels at the original resolution. Finally, we calculated the variance per-pixel with and without our results, and the ratio of the former over the later was used to assess if our maps increased (i.e., ratio ≥1) or reduced (i.e., ratio <1) the current uncertainty about the AGC changes in the literature.

### Human-influenced/non-human-influenced attribution

The dynamic part of the disaggregation model contains three components: changes in climate, forest cover, and recovery (SM, Eq. 1). The model considered no interactions among these drivers and cannot directly distinguish whether direct human activity or drought triggered a tree mortality event. Therefore, the direct attribution of the AGC changes to anthropogenic (e.g., degradation from logging or human-induced fires) or non-human (e.g., degradation from droughts) factors is not possible^26^. To overcome this limitation, our attribution analysis leveraged on available land cover maps^26^ to identify areas with and without human influence. The first category included deforestation, forest degradation, regrowth and others (i.e., agriculture), and the second category only included areas that remained undisturbed over the study period. This assumption enabled the attribution of annual AGC changes to land with or without human influence, and allowed us to summarize gross carbon gains and losses inside each category.

## Supplementary information

Supplementary Information

## Data Availability

Input data: The ERA-5 dataset of air temperature is available at (10.24381/cds.68d2bb30), and the maximum cumulative water deficit is available at (10.5281/zenodo.4903340). The L-VOD dataset can be found at the SMOS-IC website, (https://ib.remote-sensing.inrae.fr/). The Tropical Moist Forests dataset is available at (https://forobs.jrc.ec.europa.eu/TMF), and the European Space Agency CCI dataset can be accessed at (https://catalogue.ceda.ac.uk/uuid/af60720c1e404a9e9d2c145d2b2ead4e/). Output data: The reconstructed maps of annual AGC change at 100-m spatial resolution for the Amazon are available at: (10.17605/OSF.IO/HUYZS) and (10.17605/OSF.IO/EZJ6W). The datasets are also available in the European European Soil Data Centre 2.0 (ESDAC)^56^.

## Code availability

The codes used to estimate the disaggregation model can be found at https://github.com/arthfen/VOD_AGC.
