# Supplementary material for: Human influence on Amazon’s aboveground carbon dynamics intensified over the last decade
Source: Nat Commun. 2025 Jul 21;16:6681. doi: 10.1038/s41467-025-61856-1 (PMC12280014; doi:10.1038/s41467-025-61856-1)
Supplement: Supplementary file 1 — Supplementary Information [file 41467_2025_61856_MOESM1_ESM.pdf]

Supplementary information for

# Human influence on Amazon's aboveground carbon dynamics intensified over the last decade

Arthur Fendrich[1,2]*, Yu Feng[1,3], Jean-Pierre Wigneron[4], Jerôme Chave[5], Arnan Araza[6], Zheyuan Li[7], Martin Herold[8,9], Jean Ometto[10], Luiz E. O. C. Aragão[11,12], Isabel Martinez Cano[1], Lei Zhu[1,13], Yidi Xu[1], Philippe Ciais[1]

[1] Laboratoire des Sciences du Climat et de l'Environnement, UMR 1572 CEA-CNRS-UVSQ, Gif-sur-Yvette, France
[2] European Commission, Joint Research Centre (JRC), Ispra, Italy
[3] Eastern Institute for Advanced Study, Eastern Institute of Technology, Ningbo, China
[4] INRAE, Bordeaux Sciences Agro, UMR 1391 ISPA, Villenave-d'Ornon, France
[5] Centre de Recherche Biodiversite Environnement, CNRS, IRD, UPS, INPT, Toulouse, France
[6] Earth Systems and Global Change, Wageningen University and Research, Wageningen, The Netherlands
[7] School of Mathematics and Statistics, Henan University, Kaifeng, China
[8] Institute for Environmental Science and Geography, University of Potsdam, Germany
[9] Remote Sensing and Geoinformatics Section, Helmholtz GFZ German Research Centre for Geosciences, Telegrafenberg Potsdam, Germany
[10] Impact, Adaptation and Vulnerability Division, Instituto Nacional de Pesquisas Espaciais, Brazil
[11] Earth Observation and Geoinformatics Division, Instituto Nacional de Pesquisas Espaciais, Brazil
[12] Geography, University of Exeter, Exeter EX4 4PY, UK
[13] Department of Earth System Science, Ministry of Education Key Laboratory for Earth System Modeling, Institute for Global Change Studies, Tsinghua University, Beijing, China

*Correspondence to: Arthur Nicolaus Fendrich
(arthur.fendrich@lsce.ipsl.fr)

## C1. Disaggregation model

### *Statistical model formulation*

The disaggregation procedure adopted corresponds to the estimation of the parameters of a multi-scale statistical regression model. The present approach extends the non-linear mixed model and the estimation procedures proposed by [1], which originally adopted a Gaussian distribution with constant variance at the fine scale. The extension in our work consists of adding a linear predictor for the variance term of the original model, leading to a location-scale model [2] (Eqs. 1 to 5):

$$\boldsymbol{y} \sim \mathcal{N}(\boldsymbol{\mu}, \mathrm{V}\sigma^2) \quad \text{[Eq. 1]}$$
$$\boldsymbol{\mu} = \exp(\mathrm{X}\boldsymbol{\beta}) \quad \text{[Eq. 2]}$$
$$\mathrm{diag}(\mathrm{V}) = \exp(\mathrm{Z}\boldsymbol{\lambda}) \quad \text{[Eq. 3]}$$
$$\boldsymbol{\beta} \sim \mathcal{N}(\mathbf{0}, \Omega_1) \quad \text{[Eq. 4]}$$
$$\boldsymbol{\lambda} \sim \mathcal{N}(\mathbf{0}, \Omega_2) \quad \text{[Eq. 5]}$$

with **y** being the AGC (MgC/ha) at the fine scale, **μ** is the mean term, which is parameterized using a model matrix X and coefficients **β**, and the variance term consists of a variance-covariance matrix V multiplied by a variance parameter, $\sigma^2$. The V matrix is assumed to be diagonal, with elements defined by the product of a model matrix Z by the coefficients **λ**. The coefficient vectors **β** and **λ** are assumed to follow multivariate Gaussian distributions with mean **0** and variance-covariance matrix $\Omega_1$ and $\Omega_2$, respectively. The link between the fine and coarse scales (i.e., the average calculation) is made using an additional matrix A, which maps fine-scale pixels to the corresponding coarse cells where they spatially belong:

$$\boldsymbol{Y} = \mathrm{A}\boldsymbol{y} \quad \text{[Eq. 6]}$$

Because **y** is assumed to be Gaussian distributed and A is a linear transformation, the resulting model for **Y** at the coarse level will also be Gaussian distributed. However, the additional distribution assumptions brought by the variance structure demands modifications in the estimation procedure described by [1], with the global and simultaneous estimation of all model parameters using maximum likelihood being a very difficult task [3]. Since the model can also be recognized as a Generalized Additive Model for Location, Shape and Scale (GAMLSS) [3], we overcame this issue in an analogous way, by: i) appending a quadratic penalty to the likelihood derived by [1], and ii) implementing an iterative estimation method, which alternates between optimizing the mean or the variance term, while assuming the other as fixed. The resulting penalized likelihood function considers a tradeoff between approximating the coarse-scale constraints and enforcing the mathematical assumptions of the original model.

The new variance structure also leads to the problem of finding the proper variables to form the model matrix. The definition of the functional relationships for the mean and variance terms of the Gaussian distribution was made based on the following procedure. First, all variables were added to the linear predictor of the mean parameter and the model parameters were estimated assuming constant variance. This procedure corresponded to the implementation proposed by [1]. However, residual analysis (described in the next sections) showed the inadequacy of such assumptions for the VOD data used in the present work. Therefore, the second step consisted of performing a residual analysis at the coarse level to detect systematic biases of the results with respect to the explanatory variables. This procedure allowed us to detect which variables could provide relevant information for the variance term. Then, in a third step, the relevant variables were added to the linear predictor of the variance term, and model parameters were re-estimated.

The formulation adopted for Eqs. 1 to 5 allows the incorporation of multiple basis-penalty smoothers [2]. In the final model adopted, the functional relationship between model parameters and explanatory variables can be described, using common notation for additive models, as:

$$\mu_{i,t} = \exp\Bigg[\overbrace{s_1(x_i, y_i) + s_2(AGC_{i,2010})}^{\text{Static effects}} + \overbrace{\underbrace{\sum_{n=(t-3)}^{t} s_3(T_{i,n}, D_{i,n})}_{\text{Climate}} + \underbrace{s_4(M_{i,t})}_{\text{Forest cover}} + \underbrace{\mathbf{1}_{u_{i,t}} s_5(L_{i,t}) + \mathbf{1}_{d_{i,t}} s_6(L_{i,t}) + \mathbf{1}_{r_{i,t}} s_7(L_{i,t})}_{\text{Recovery}}}^{\text{Dynamic effects}}\Bigg]$$

$$\text{diag}(\text{V})_{i,t} = \exp\left[s_8(x_i, y_i) + s_9(M_{i,t})\right]$$

with $\mu_{i,t}$ being the AGC of the pixel indexed i at year t; $x_i$ and $y_i$ being the latitude and longitude, respectively; $AGC_{i,2010}$ being ESA's CCI AGB information for 2010; $T_{i,n}$ and $D_{i,n}$ being the 4-year time-series of maximum temperature and water deficit, respectively; $M_{i,t}$ being the forest cover class reclassified into five categories: 'undisturbed', ‘regrowth’, ‘degraded’, ‘deforested’, and, ‘other land cover'; $L_{i,t}$ being the time since last disturbance; and $1_u$, $1_d$, $1_r$ being indicator functions for the presence of ‘undisturbed’, ‘degraded’ and ‘regrowth’ pixels, respectively. The function exp(x) is the link function that makes median AGC to be a positive quantity, $\text{diag}(V)_{i,t}$ is the diagonal element of V correspond to pixel i at year t, and $s_k(.)$, k = {1, 2, 3, 4, 5, 6, 7, 8, 9}, are smoothers estimated from the data.

The final functional form described above was defined based on the physical understanding of the process of AGC change. The smoothers $s_1$ and $s_2$, called static effects, represent the long-range autocorrelation not represented by the other explanatory variables and the initial state of the AGC stocks provided by an external source, respectively. Among the dynamic effects: $s_3$ represents the time-series of interactions between temperature and water deficit, which can affect photosynthesis rates or induce forest degradation by drought and fires, for example; $s_4$ captures the effect of changes among forest cover types on AGC stocks; and $s_5$, $s_6$ and $s_7$ represent the AGC dynamics after land cover change. The term $s_5$ only applies to undisturbed forests and attempts to capture temporal variations in the dynamics of intact forests that can not be captured by the spatial resampling of climate data to the fine scale. Smoother $s_6$ applies to degraded forests, and attempts to capture the AGC gain due to the regrowth of trees after degradation. Analogously, $s_7$ applies to regrowth pixels and attempts to capture the trajectory of AGC after forest plantation. In the variance parameter, $s_8$ attempts to capture short-range unmodeled autocorrelation, and $s_9$ represents variations in model uncertainty after land cover change.

In the mean parameter, the smoothers used were cubic splines for univariate variables or tensor products of cubic splines for multivariate variables [2]. A basis dimension of 12 was adopted for latitude, longitude, AGC and temperature. Even though the forest cover dataset would be better modeled as a factor, we opted to replace it by cubic splines for practical reasons. The basis dimensions for forest management and the recovery curves were 4 and 8, respectively. In the variance parameter, $s_8$ was assumed to be a Gaussian process with a Matérn covariance function [4] and, for $s_9$, cubic splines with basis dimension 4 was adopted.

### Further considerations

The disaggregation problem has a multi-scale nature, and representing the link between the fine and coarse levels creates an additional constraint during model formulation. In order for calculations to be tractable, the aggregation of the random variables at the fine level should lead to a known distribution at the coarse level. This means that, unlike regular GAMLSS models [3], the choice of a distribution for the data is constrained. Few distributions respect this property, including the Gaussian, Cauchy, Gamma (with a constant rate parameter), and Lévy distributions. On the one hand, the drawback of the first two distributions is that they are defined over the set of real numbers while AGC is a non-negative quantity. On the other hand, the drawback of the last two distributions is the constant rate parameter of the Gamma distribution, which is unlikely to be a realistic assumption, and the variable support of the Lévy distribution, which makes the estimation procedure highly unstable. Therefore, several tests were performed to choose between these four options. The results of tests led to discarding the Gamma, Cauchy and Lévy distributions due to poor residual diagnostics and long number of iterations needed to reach convergence, which could lead to prohibitively high computing times. Thus, to minimize the fundamental problem with the unbounded support of the Gaussian distribution, residual analysis was performed to assess model fit and explore potential biases.

### Residual analysis

As discussed previously, the model resulting from Eqs. 1 to 5 and the transformation of Eq. 6 has several basic assumptions that have to be properly checked for their adequacy using residual diagnostics. These assumptions include the distributional assumptions for the response variable, the inclusion of explanatory variables in the mean and variance terms, among others. Even though such analysis can not be performed at the fine level due to the lack of observations, the diagnostics using coarse information is still helpful to indicate whether the aggregated result of all model assumptions made is suitable for the data used.

Given the non-constant variance introduced in the formulation, raw residuals (i.e., observed minus fitted values) may fail to capture part of the assumed model structure, and other options are preferred. The most promising alternative in this case is normalized quantile residuals (hereinafter referred to as 'residuals' unless mentioned otherwise), introduced by [5], which are default for the diagnostics of GAMLSS models [3]. These residuals are calculated by first using a probability integral transform of the VOD data given the fitted distribution, followed by an application of the inverse cumulative distribution function of a standard Gaussian distribution to the result. When model assumptions are adequate to the data, residuals should follow a standard Gaussian distribution.

Thus, the diagnostics consisted of generating quantile-quantile plots (i.e., qq-plots) and visually assessing the deviation between the quantiles of model residuals and those of a standard normal distribution. An overall analysis was made by pooling all residuals (i.e., from all grid cells at all years) together, and several separate analyses were made by splitting residuals according to the explanatory variables used in the model. Because this procedure was performed at the coarse scale, the later analyses used the average values of the continuous predictors (e.g., the average temperature or water deficit) and the total fraction of categorical predictors (e.g., the fraction of undisturbed fine-scale pixels).

The overall residual diagnostics is presented in Fig.S1 (left). It indicates an adequate fit, with few deviations to the 1:1 line of theoretical quantiles of a standard Gaussian

distribution. When model residuals are split according to the percentiles of the predicted median values (Fig.S1, right), the results are still close to the diagonal red line in most cases. The most severe deviations occur for the class of lowest and highest predicted median values (i.e., “< 11.1%” and “> 88.8%”, respectively), a pattern that seemed persistent in all tests performed during model fit.

**Fig.S1. Model residual diagnostics. Results for all cells pooled together (left) and split by the percentiles of the predicted median values (right).** In the right figure, predicted median values were divided into 9 classes, each containing an equal proportion of the data. The notation Q1 refers to the lowest one-ninth of median values, while Q2 refers to the values between the 11.1% and 22.2% percentiles, and so on.

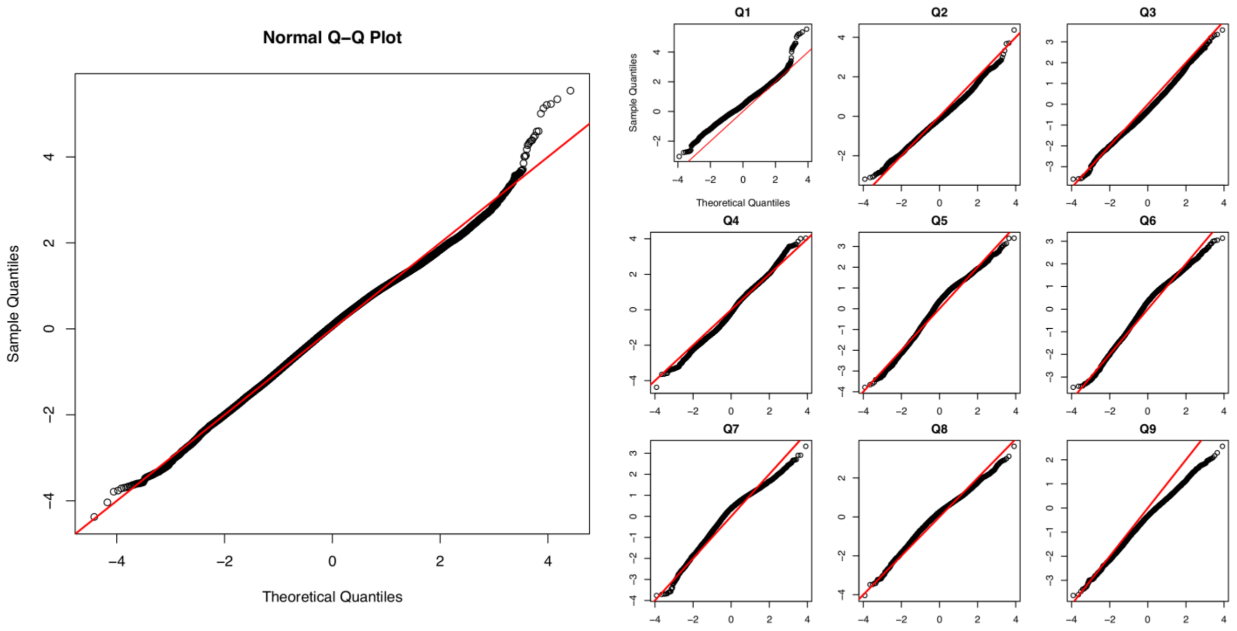


The validation split by percentiles of the explanatory variables showed a similar pattern for all variables included in the model. For this reason, only the temperature and fraction of undisturbed forests were selected to be presented in Fig.S2 (top and bottom, respectively). The plots indicate a good fit to all percentiles of the temperature data, and for most of the fractions of undisturbed forests. In cells with many unobserved forests (i.e., fraction Q8), some deviation is present, but the pattern does not depart much from the 1:1 line. These indicate a lack of bias in the model fit with respect to the model variables.

**Fig.S2. Model residual diagnostics split according to the percentiles of the explanatory variables: temperature (top) and fraction of undisturbed forests (bottom)**. The notation Q1 refers to the lowest one-ninth values of temperature (top) or fraction of undisturbed forests (bottom), while Q2 refers to the values between the 11.1% and 22.2% percentiles, and so on.

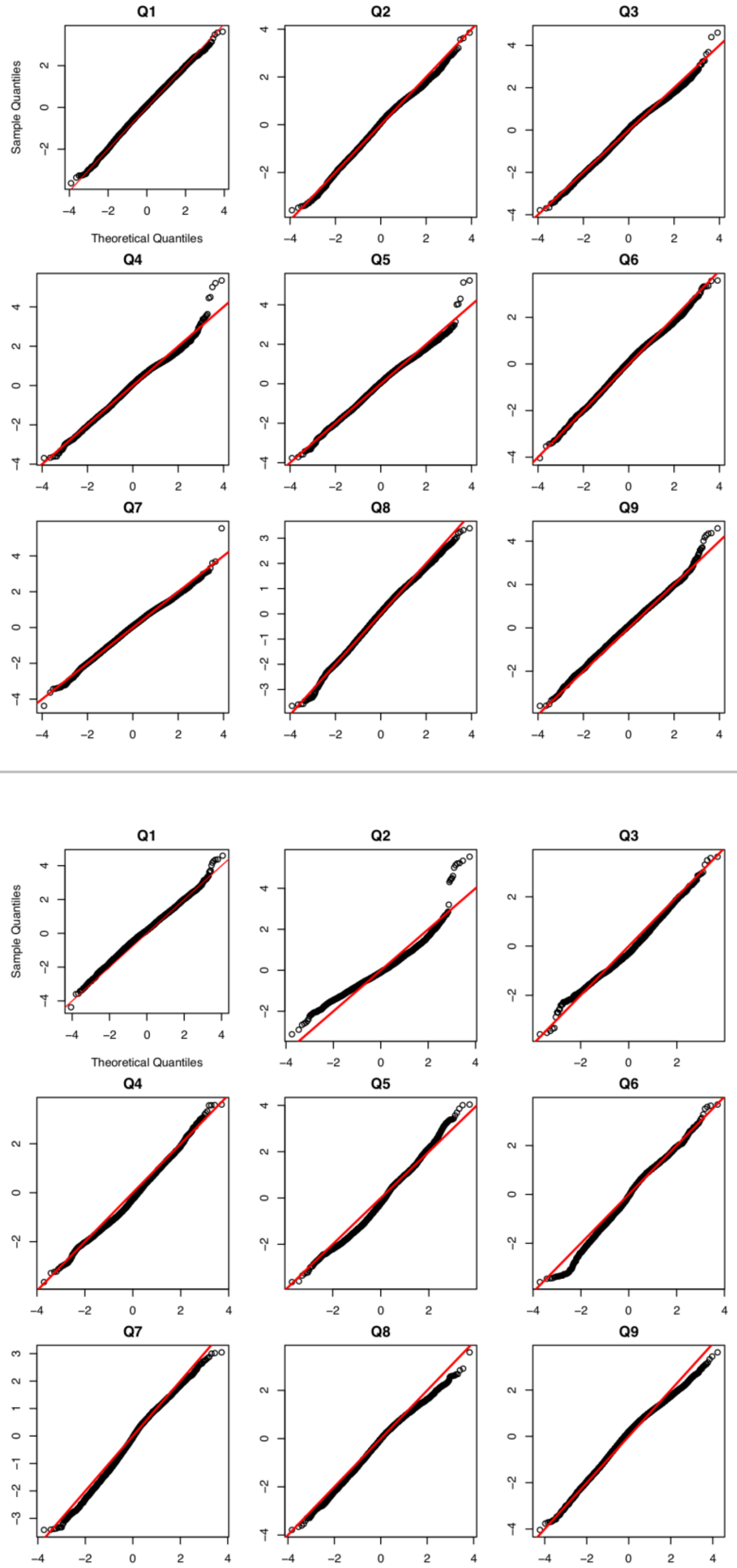


As mentioned above, the qq-plots presented assess whether the distributions obtained at the coarse level adequately represent the VOD data observed. While this is useful for model diagnostics, users may be interested in using only the expected values for their calculations. In that case, a plot of the actual VOD AGC versus the fitted expected

values could be relevant. Such information is presented in Fig.S3, where it can be seen that values are generally spread around the 1:1 line, with correlation coefficients above 0.94, and root mean square errors (RMSE) around 12.6 MgC ha$^{-1}$.

**Fig.S3. Plot of actual (i.e., VOD) versus reconstructed values of AGC: all years pooled together (top) and separate years (bottom)**.

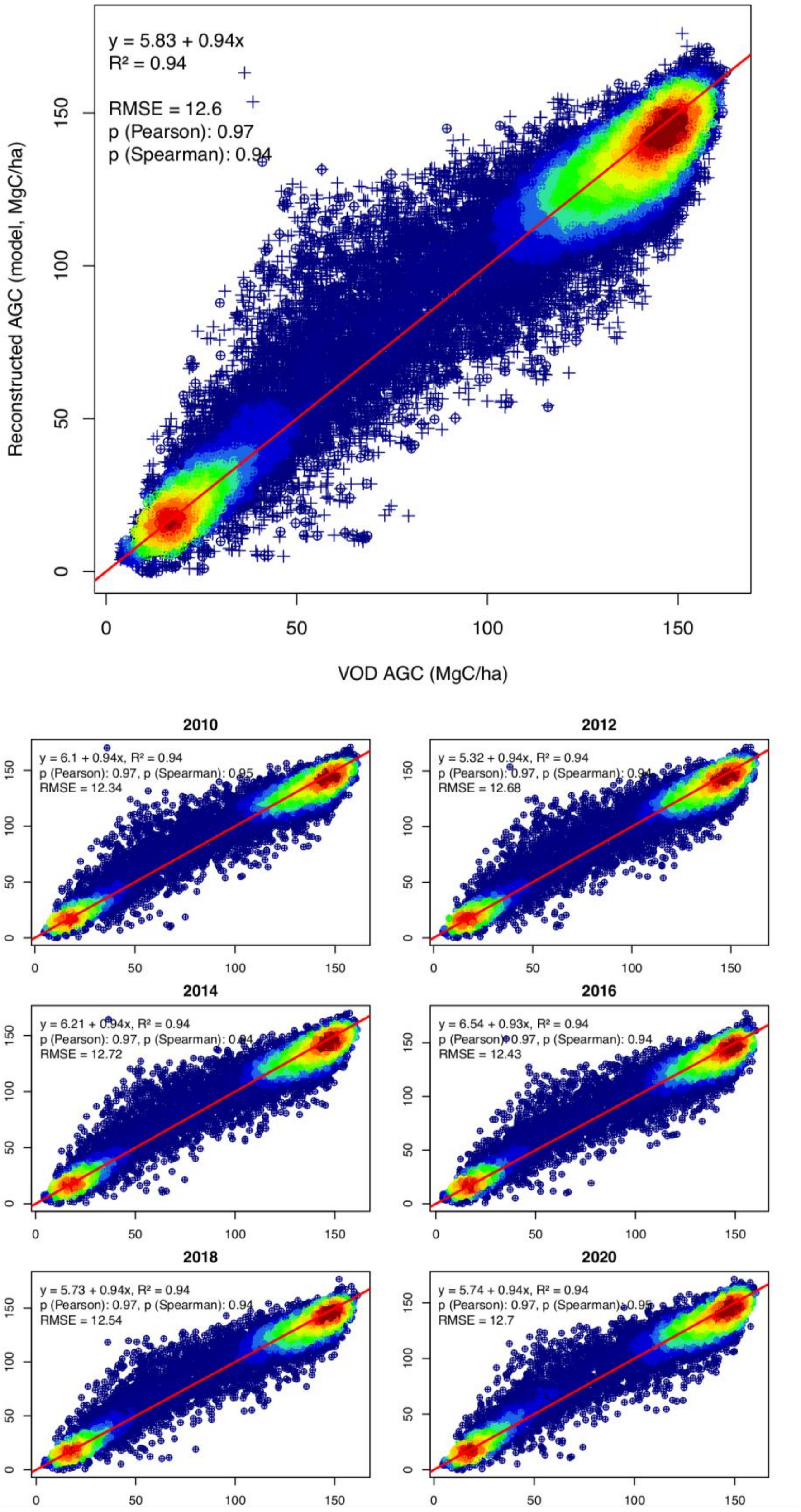

### *Estimated smoothers*

Another model diagnostics consisted of visually inspecting the estimated effects of each smoother in the functional form of the model previously described. These plots can be interpreted to understand how the relationships obtained relate to the previous knowledge of the physical understanding of the process of AGC change. Fig.S4 shows results for the first three smoothers, $s_1$, $s_2$, and $s_3$.

Overall, they have a relatively similar order of magnitude, indicating no evident domination of one over the others. $S_1$, which captures long-range autocorrelation, suggests a pattern of lower values unexplained by the other explanatory variables around the arc of deforestation in Brazil and a pattern of higher values near the Andean region. The standard deviation of $s_1$ is relatively low across the study area, indicating a stable fit. $S_2$ includes an external aboveground biomass (AGB) map as an explanatory variable, and despite some wiggliness, shows a reasonable pattern of increased contribution to the linear predictor as the AGB values increase. In $s_3$, where the interaction between temperature and maximum cumulative water deficit (MCWD) is modeled, interpretation is more easily made in the range of temperature between 300 and 305K (i.e., 26.85 to 31.85 °C), where most combinations are located. In that region, higher MCWD leads to a negative effect, indicating the possible loss of AGC due to droughts. Such a pattern, however, is not present in colder temperatures below 300K (i.e., 26.85 °C), where few points are located. Since a time series of four years is used for $s_3$ and Fig.S4 shows only the example of one year, the range of values estimated (i.e., from -0.2 to 0.2) must also be multiplied by four for the comparison with other smoothers.

**Fig.S4. Smoothers estimated from the disaggregation model: $s_1$, $s_2$, and $s_3$.**

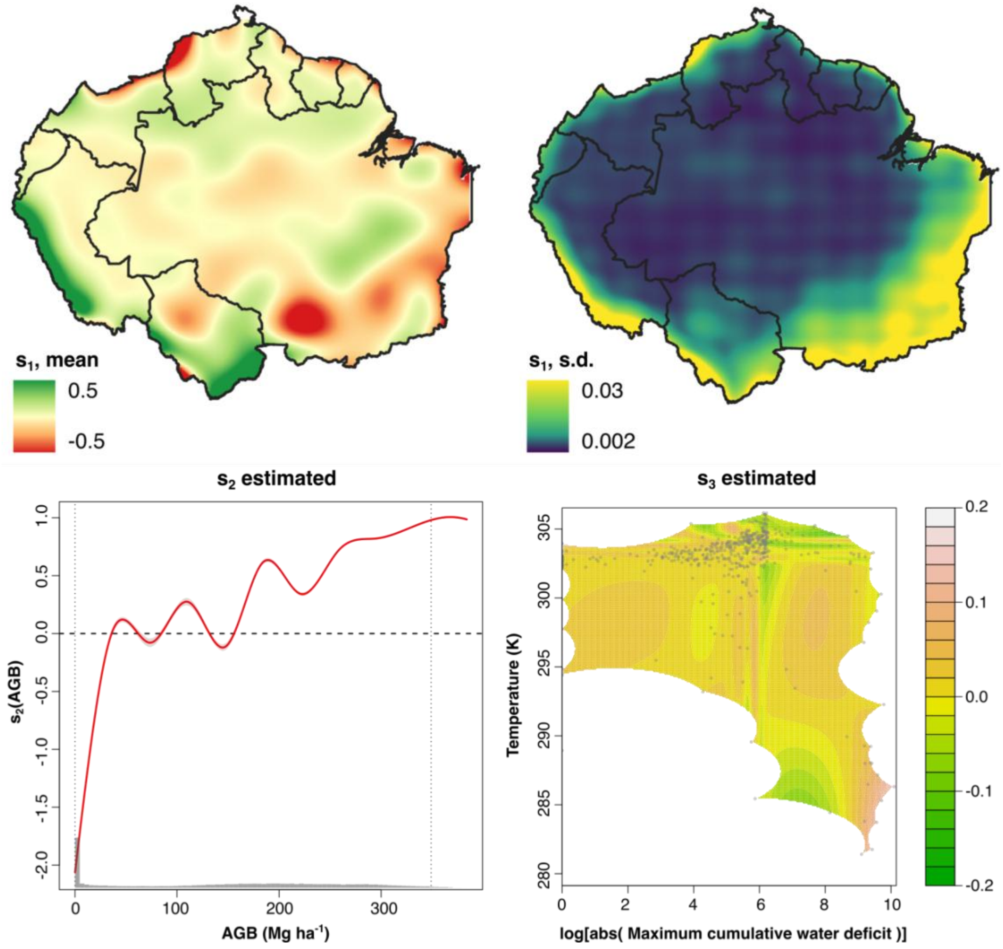

The last four smoothers, $s_4$, $s_5$, $s_6$ and $s_7$, are displayed in Fig.S5. The land cover smoother, $s_4$, shows a reasonable increase of the estimated values across deforested, disturbed, other (i.e.,, agriculture), regrowth and undisturbed areas, respectively. Such a pattern indicates that deforestation is always a loss in the model, regardless of the previous land cover type. The regrowth class has higher uncertainty due to its relatively small number of pixels in the landscape. In $s_5$, where the fluctuations in undisturbed forests are represented, the vertical scale shows a much lower effect than all other smoothers, indicating that most of the temporal variation in undisturbed forests was properly captured by the other temporal elements in the model. The histogram in the bottom of $s_5$ shows a balanced number of pixels in age classes.

The pattern of $s_6$ is likely the only one that does not fully correspond to the physical understanding of forest regrowth, with the decreasing pattern after four years since disturbance pointing to a loss of AGC after half a decade of forest regrowth. While this is probably influenced by the small number of observations in these classes, shown in the histogram at the bottom of $s_6$, more reasons are discussed in the *C6. Limitations* section of this document. Finally, in $s_7$, a reasonable pattern of increase is shown, indicating the recovery of forests from past degradation events.

**Fig.S5. Smoothers estimated from the disaggregation model: $s_4$, $s_5$, $s_6$, and $s_7$.**

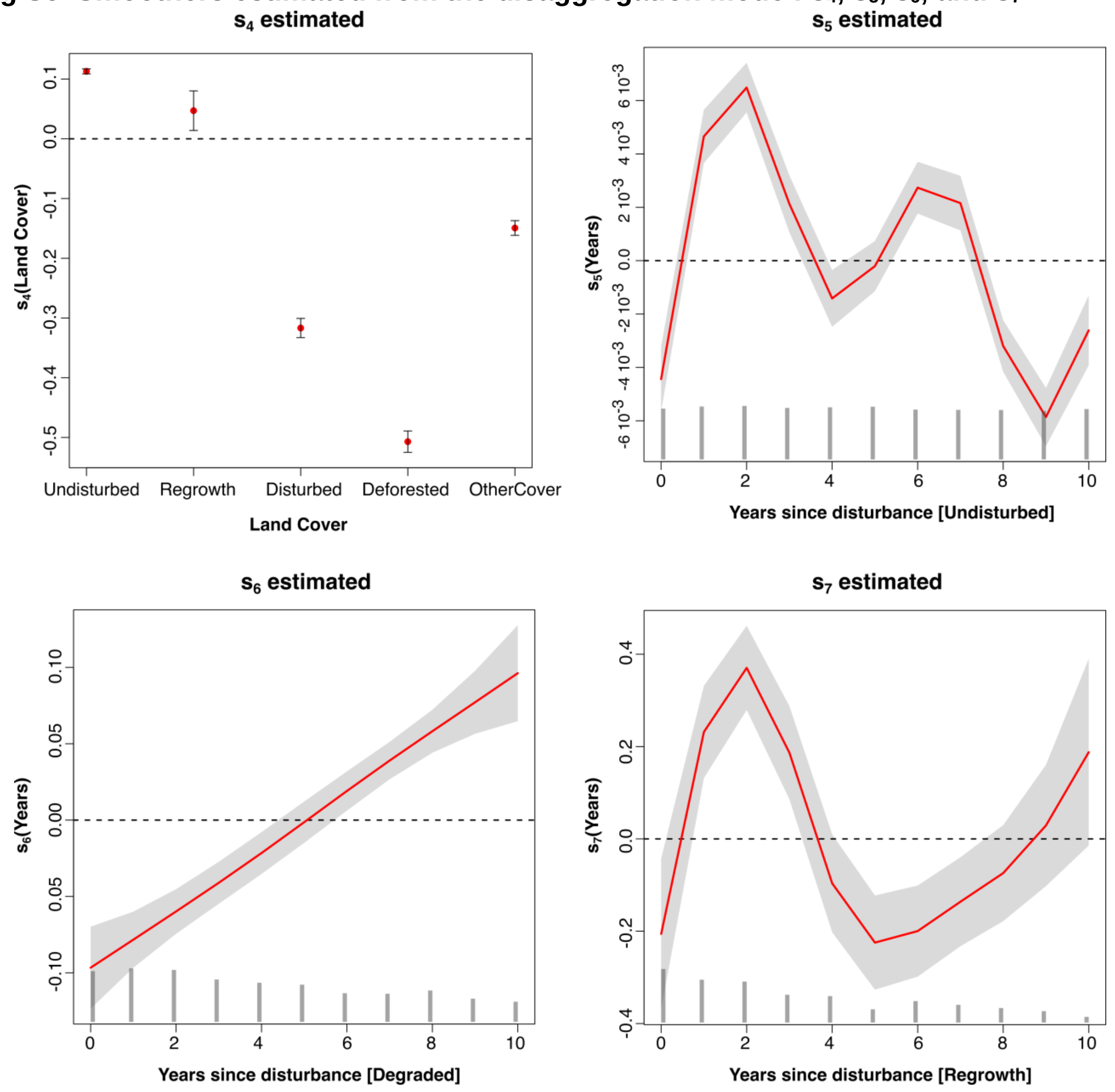

## C2. Comparison of AGC stocks against plot data - Araza et al. (2022)

The AGC estimates are based on forest inventory data from monitoring and research plots distributed across the Amazon. Individual AGC was estimated based on trunk diameter and tree height measurements using allometric relationships and later aggregated for all individuals to upscale AGC at the plot level. The location of the forest inventory data is displayed as red dots in Fig.S6, and comparisons are shown in Fig.S7.

**Fig.S6. Disaggregated aboveground carbon stocks at the 0.00089° (~ 100m) spatial resolution: mean (top) and lower bound of the standard deviation (bottom).** Values correspond to the year of 2020. Red dots correspond to the reference data used for model validation at a 0.1° (~ 10km) spatial resolution, and a region with human activity is shown in detail (top close-ups). The lower bound of the standard deviation can be used to calculate confidence intervals for the mean values.

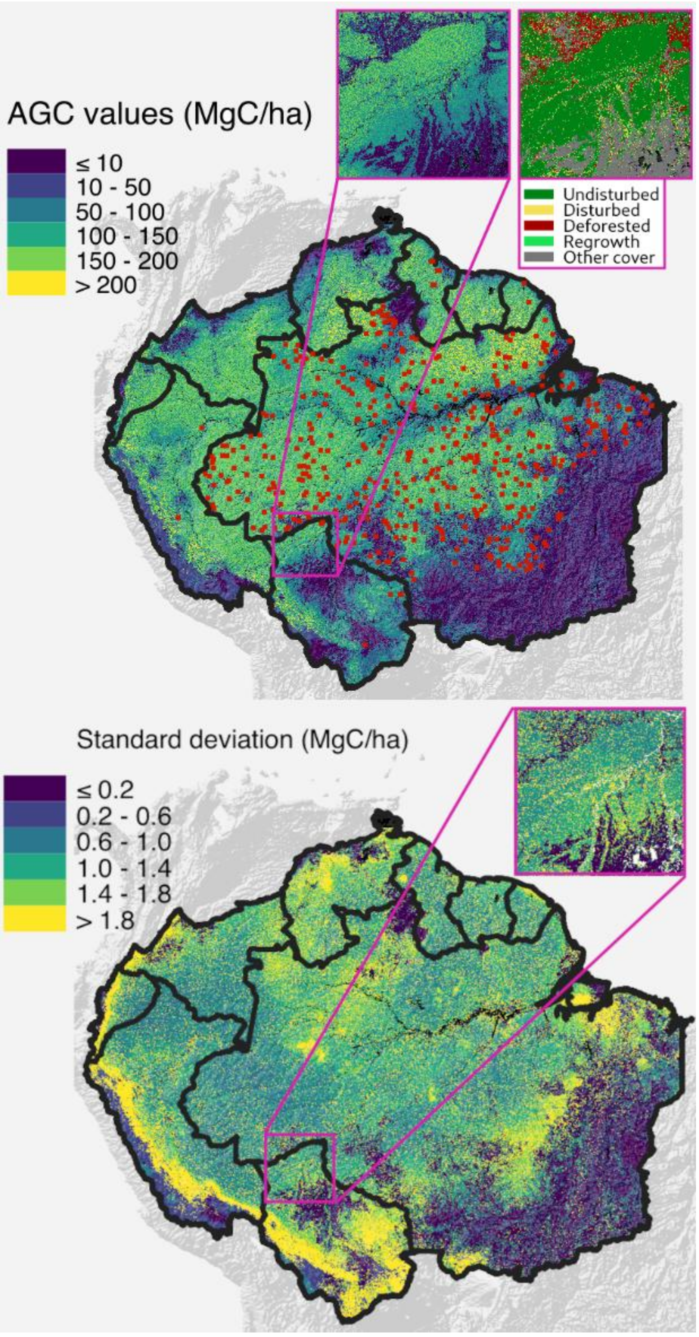

**Fig.S7. Comparison of different AGC maps against reference plots at a 0.1° spatial resolution.** a-b: CCI biomass map, version 4 [6] (**a**), JPL map [7] (**b**). The red line is a 1:1 line.

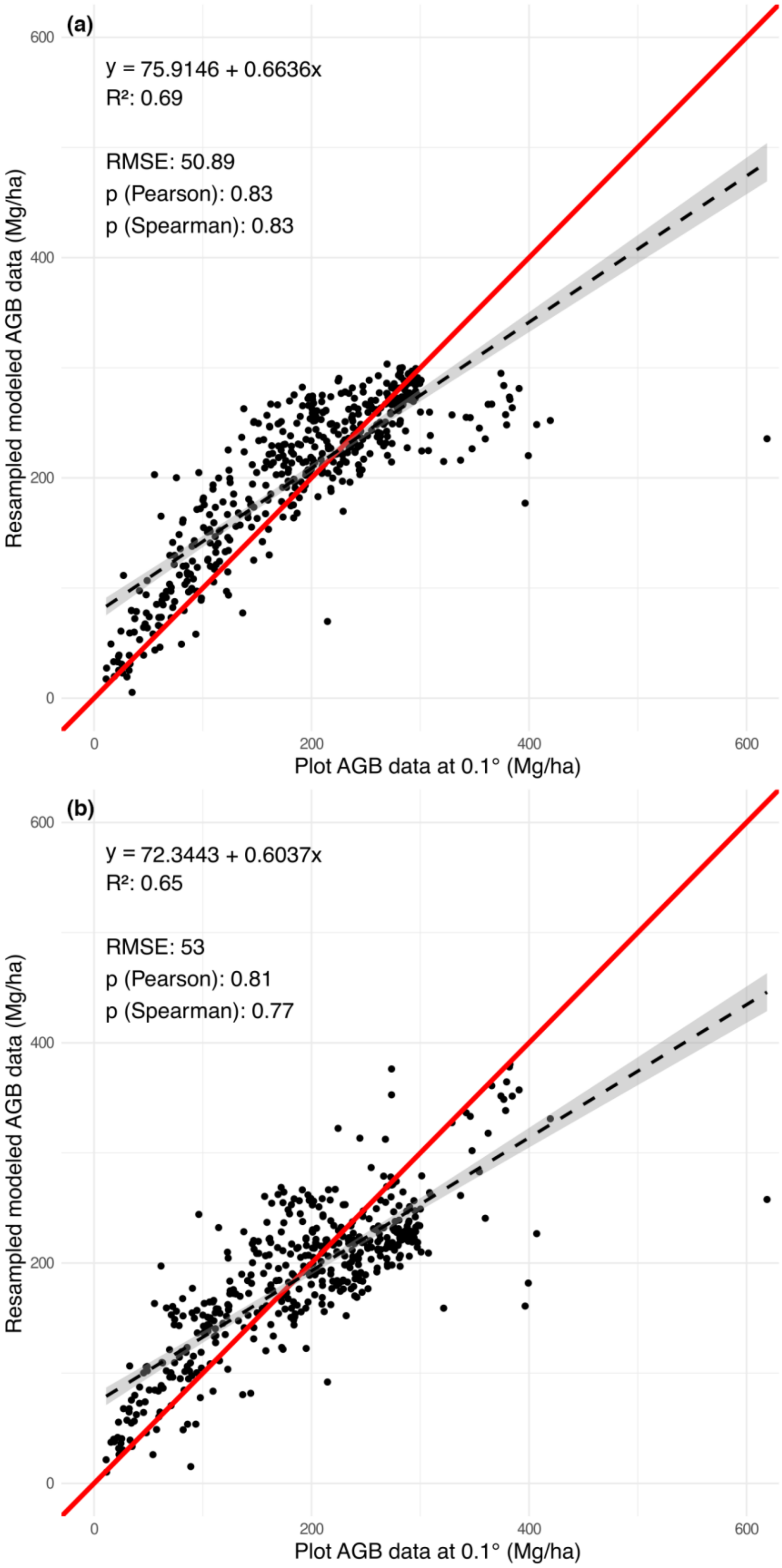

### C3. Comparison of AGC change against plot data - Hubau et al. (2020) [8]

Brienen et al. (2015) [9] and Hubau et al. (2020) [8] estimated the net carbon sink based on tree census data from intact forest plots in the Amazon. They separately reconstructed carbon gains and losses based on estimates of tree growth and recruitment, and mortality to estimate the net carbon sink. Two comparisons were made against their plot observations of AGC change. These results can only be obtained with the datasets that provide yearly estimates of AGC, so the current model and the JPL dataset [7] were used. The two comparisons were:

1. The first, called *S1,* consisted of filtering the database to keep only the data collected during a period that overlaps the current study (i.e., 2010-2020). In this case, the map corresponding to the year of observation was used. This procedure led to n = 89 observations.
2. The second, called *S2*, does not filter the database, using n = 596 and including older observations. That was possible with an additional assumption that if a plot was visited between years Y1 and Y2, the map adopted for the first year of observation was 2010, and the last year was 2010 + (Y2 - Y1). As in Yang et al. (2023), the last year was not allowed to be greater than 2014 since no El Niño events are captured in the Hubau et al. (2020) database.

Most coordinates from the database were precise to one or two decimal digits, leading to large uncertainties in their actual location. To overcome this problem, each observation's latitude and longitude were considered a random variable with a uniform distribution centered in the coordinates provided, ranging from 50% to the most precise coordinate digit. For example, a coordinate of -1.43° was considered to vary between -1.435° and -1.425°. The calculations were performed 100 times by sampling from these uniform distributions, and only pixels falling into undisturbed areas were considered. These calculations were used to compare the averages of modeled and observed data, and one of these results was stored for visualization.

Fig.S8 presents the results for S1 (left) and S2 (right), showing some overlap between the distributions. In S1, the data average and median were 0.04 and 0.11 MgC $ha^{-1}$ $yr^{-1}$, respectively, while the model average and median were 0.95 ± 0.08 and 0.58 ± 0.05 MgC $ha^{-1}$ $yr^{-1}$, respectively, and the JPL average and median were 1.60 ± 0.05 and 0.44 ± 0.006 MgC $ha^{-1}$ $yr^{-1}$, respectively.  In S2, the data average and median were 0.34 and 0.60 MgC $ha^{-1}$ $yr^{-1}$, respectively, while the model average and median were 0.44 ± 0.02 and 0.43 ± 0.02 MgC $ha^{-1}$ $yr^{-1}$, respectively, and the JPL average and median were 1.29 ± 0.03 and 0.30 ± 0.05 MgC $ha^{-1}$ $yr^{-1}$, respectively.

**Fig.S8. Comparison of results against the plot data of Hubau et al. (2020) for the filtered database (i.e. S1, left) and unfiltered database (i.e. S2, right).** Units are MgC/ha**.**

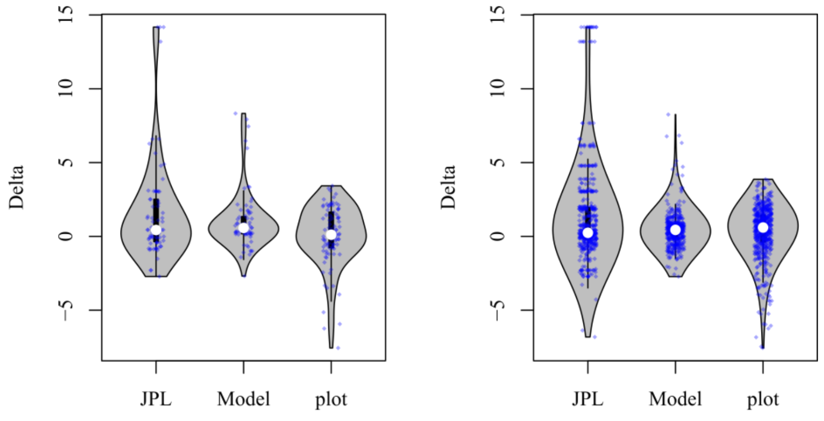

## C4. Comparison of AGC change against VOD data

Another comparison performed at a different level was an evaluation of how the AGC change from 2010 to 2018 is represented on other published datasets. Since such an analysis does not demand yearly AGC values, the maps used were those produced by JPL [7], CCI [6], WRI [10, 11] and our model. The comparison was performed by first re-aggregating the aforementioned datasets to the 0.25° spatial resolution, then plotting their AGC variation in the period against the same variation in the VOD dataset. The results of Fig.S9 show that, while our model presents a positive linear trend against the AGC variation of the VOD data due to the calibration of the disaggregation procedure, other data sources show less consistent patterns. The plot for the CCI dataset presents a bimodal density, with the first peak around the 1:1 line and the second peak above it. The plot for the JPL dataset presents a nearly flat regression line, indicating a tendency to flatten the changes observed with VOD. Compared to the others, the points in the WRI plot are more scattered and less concentrated around the 1:1 line, leading to a linear regression line of negative slope. Fig.S10 presents the same results in the form of maps.

**Fig.S9. Comparison of AGC change between 2010 and 2018 of four different models against VOD data.** The continuous black line is a 1:1 line, while the dashed black line shows the output of a linear regression model.

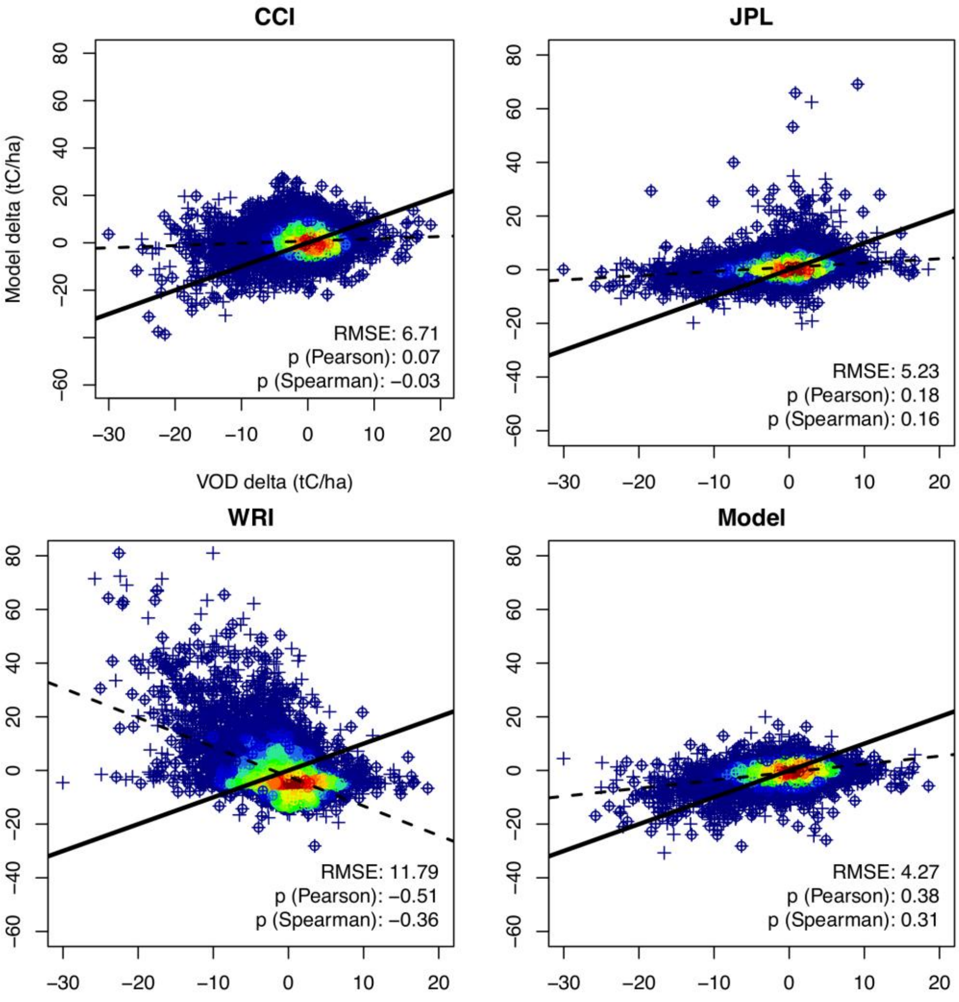

**Fig.S10. Maps comparing the AGC change between 2010 and 2018 for four different models against VOD data.** The maps display the difference between the AGC change calculated with a given model minus the same quantity calculated with VOD data.

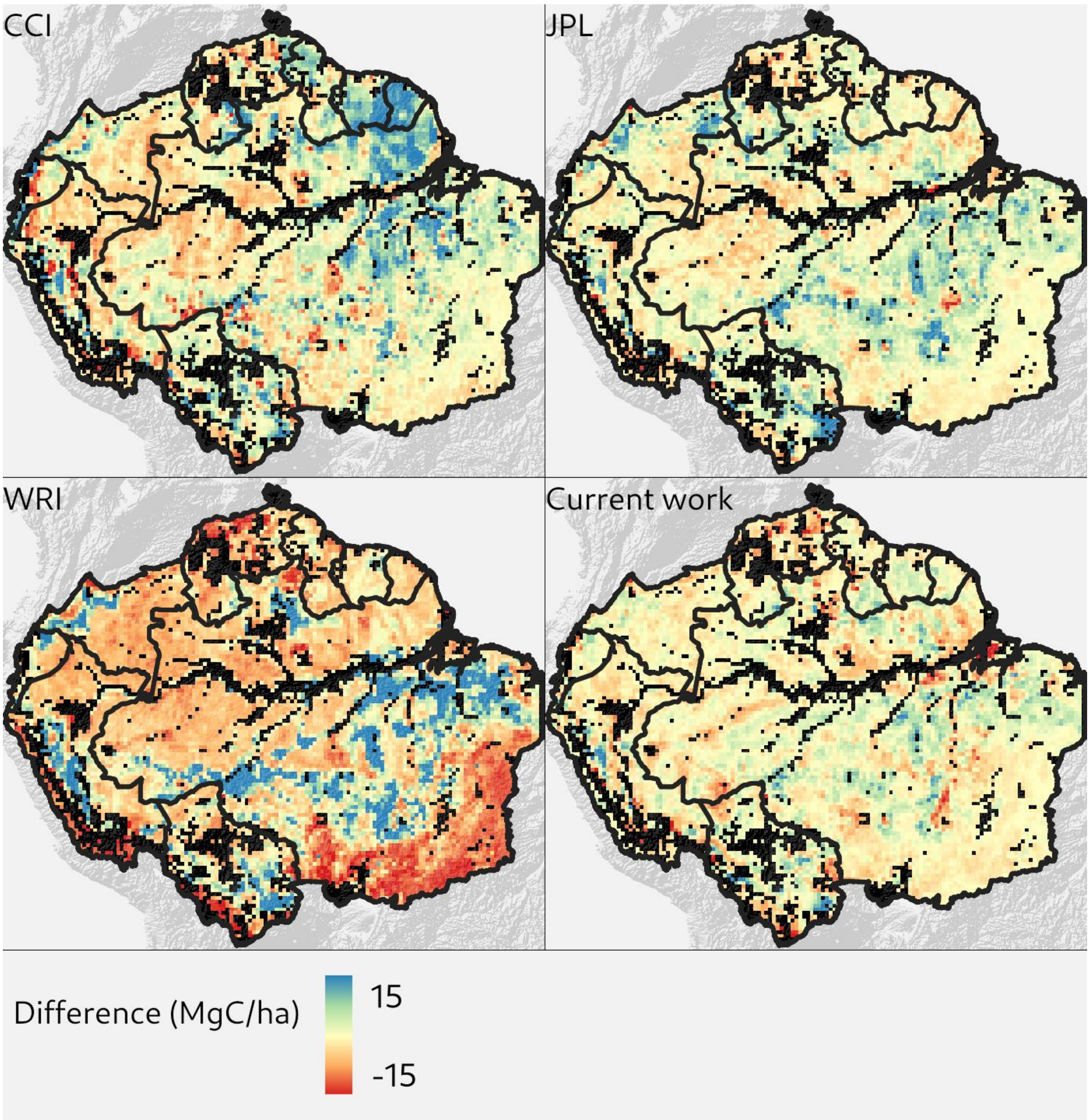


## C5. AGC validation at a finer scale

The comparisons presented so far (i.e., Fig.S7, Fig.S8, Fig.S9, Fig.S10) require the pre-processing step of re-aggregating our results to a coarser resolution to allow a harmonization across scales. Although useful, such evaluations do not allow a proper comparison of the model performance at the 100m spatial resolution for which the disaggregation was made. To overcome this issue, the two products available at a 100m spatial resolution (i.e., our maps and ESA CCI) were compared against the largest Light Detection and Ranging (LiDAR) database ever collected over the Brazilian Amazon, by Ometto et al. (2023) [17]. The model result and the CCI map for 2017 were used and two comparisons were made: at 250m and 50m spatial resolution.

### C5.1 Comparison at a 250m spatial resolution

The first comparison concerned the dataset made publicly available by Ometto et al. (2023) [17], of 148,013 pixels with available LiDAR information at 250m spatial resolution, a dataset that corresponds to the training set of the random forest model used by Ometto et al. (2023). The comparison was then made in two steps. First, a polygon of 250 x 250m was drawn by considering the coordinates provided as their centroids. Then, the average AGC from the pixels of our map and CCI within each polygon was calculated. A factor of two to convert between Mg $ha^{-1}$ and MgC $ha^{-1}$ was adopted when needed, and the most frequent land cover class was considered as predominant. When there was no two or more predominant land cover classes, one was sampled and randomly assigned. The results are presented in Fig.S11 and Fig.S12, and a summary of comparisons is shown in Table S1.

**Fig.S11. Assessment of AGC with LiDAR data (250m).** The plots compare the results of our model (left) and of the CCI dataset (right) with AGC estimates at 250m spatial resolution from Ometto et al. (2023). Each row details the comparison based either on all (a,d), undisturbed (b,e) or disturbed (c,f) pixels.

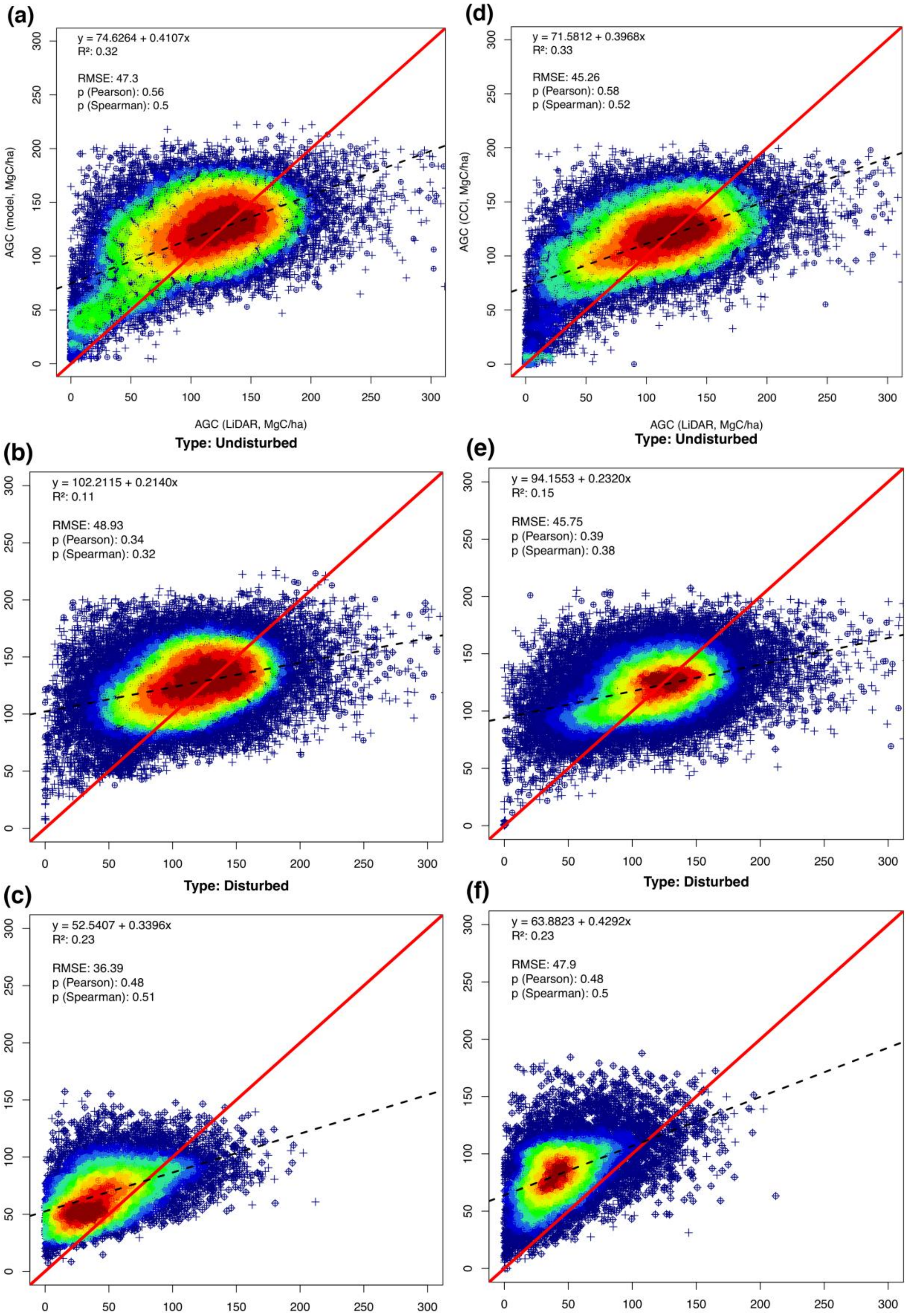

**Fig.S12. Assessment of AGC with LiDAR data (250m).** The plots compare the results of our model (left) and of the CCI dataset (right) with AGC estimates at 250m spatial resolution from Ometto et al. (2023). Each row details the comparison based either on deforested (a,d), regrowth (b,e) or other cover (c,f) pixels.

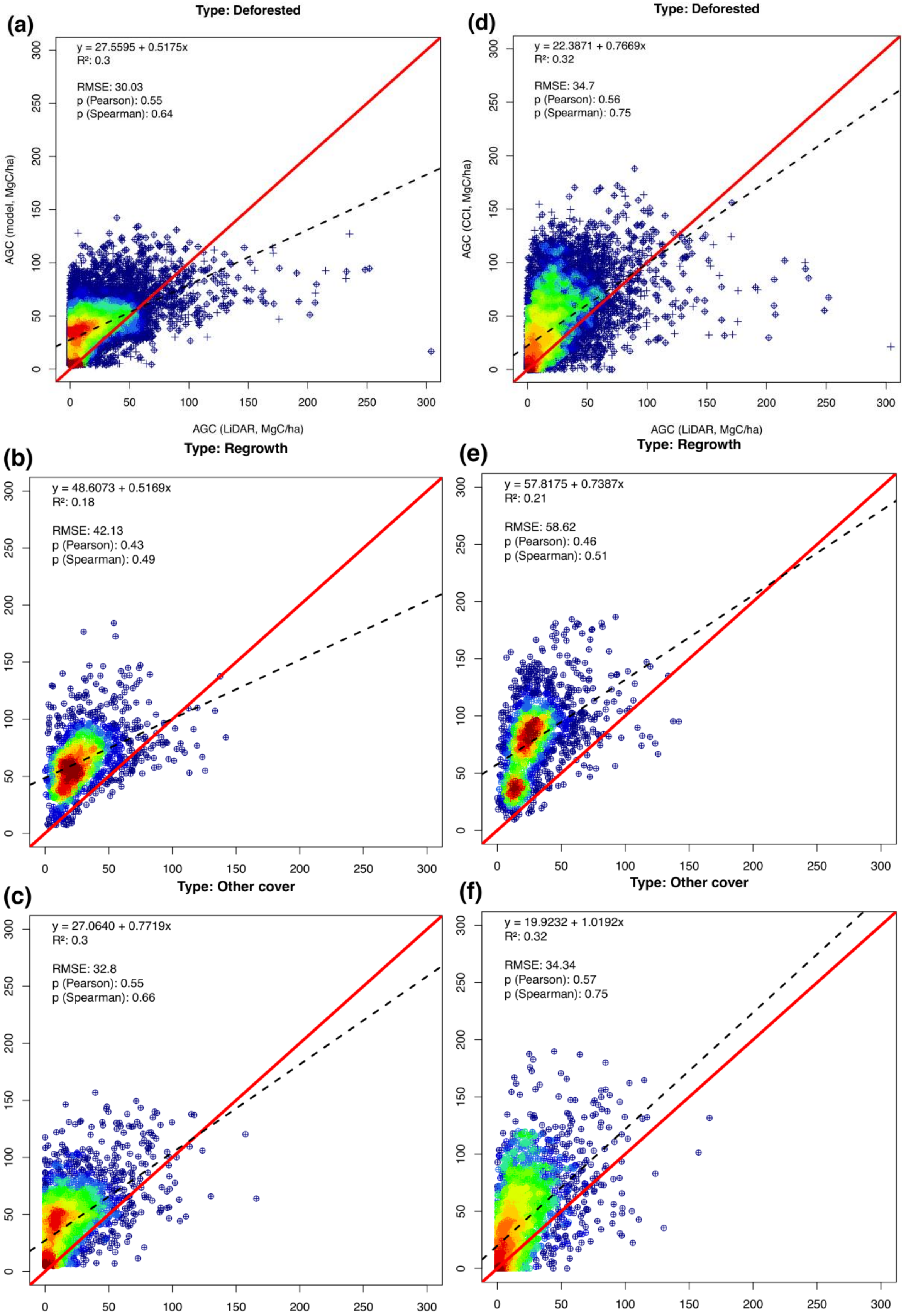

**Table S1. Summary of the validation of our model and CCI against the pixels of Ometto et al. (2023) with LiDAR information, at a 250m spatial resolution.** To facilitate comparison, the best values are highlighted in green.

| Pixels considered | Root mean square error (RMSE) | Pearson correlation coefficient | Spearman correlation coefficient | Coefficient of determination ($R^2$) |
|---|---|---|---|---|
| All pixels | Our work: 47.30<br>**CCI: 45.26 (-4.3%)** | Our: 0.56<br>**CCI: 0.58** | Our: 0.50<br>**CCI: 0.52** | Our: 0.32<br>**CCI: 0.33** |
| Undisturbed only | Our: 48.93<br>**CCI: 45.75 (-6.5%)** | Our: 0.34<br>**CCI: 0.39** | Our: 0.32<br>**CCI: 0.38** | Our: 0.11<br>**CCI: 0.15** |
| Disturbed only | **Our: 36.39 (-24.0%)**<br>CCI: 47.90 | Our: 0.481<br>**CCI: 0.484** | **Our: 0.515**<br>CCI: 0.499 | Our: 0.232<br>**CCI: 0.235** |
| Deforested only | **Our: 30.03 (-13.5%)**<br>CCI: 34.70 | Our: 0.55<br>**CCI: 0.56** | Our: 0.64<br>**CCI: 0.75** | Our: 0.30<br>**CCI: 0.32** |
| Regrowth only | **Our: 42.13 (-28.1%)**<br>CCI: 58.62 | Our: 0.43<br>**CCI: 0.46** | Our: 0.49<br>**CCI: 0.51** | Our: 0.18<br>**CCI: 0.21** |
| Other cover only | **Our: 32.80 (-4.5%)**<br>CCI: 34.34 | Our: 0.55<br>**CCI: 0.57** | Our: 0.60<br>**CCI: 0.75** | Our: 0.30<br>**CCI: 0.32** |

Overall, our model and CCI have a similar performance when compared against the LiDAR dataset at a 250m spatial resolution. CCI has a stronger linear relationship in all cases except the Spearman correlation for disturbed forests, as evidenced by the higher correlation coefficients and model $R^2$. A direct comparison of values indicates an RMSE 4.3% lower for CCI when all pixels are pooled together or 6.5% lower when only undisturbed forests are considered. For disturbed, deforested, regrowth, and other cover pixels, our model presented RMSEs lower by 24.0%, 13.5%, 28.1%, and 4.5%, respectively.

### C5.2 Comparison at a 50m spatial resolution

In the second round of review of the current work, the authors were granted access to the original database used by Ometto et al. (2023) before the re-aggregation to a 250m spatial resolution. The total database consists of 2,253,598 points. Therefore, a second comparison was made by following three steps. First, all 50m LiDAR points were assigned to one pixel of the 100m resolution maps of our results and CCI 2017. Next, when multiple LiDAR points were within the same 100m pixel, they were averaged, which reduced the number of points to around 640,000. Finally, the results of the previous step were compared against our results and CCI 2017. The results are presented in Fig.S13 and Fig.S14, and a summary of comparisons is shown in Table S2.

**Fig.S13. Assessment of AGC with LiDAR data (50m).** The plots compare the results of our model (left) and of the CCI dataset (right) with AGC estimates at 50m spatial resolution from Ometto et al. (2023). Each row details the comparison based either on all (a,d), undisturbed (b,e) or disturbed (c,f) pixels.

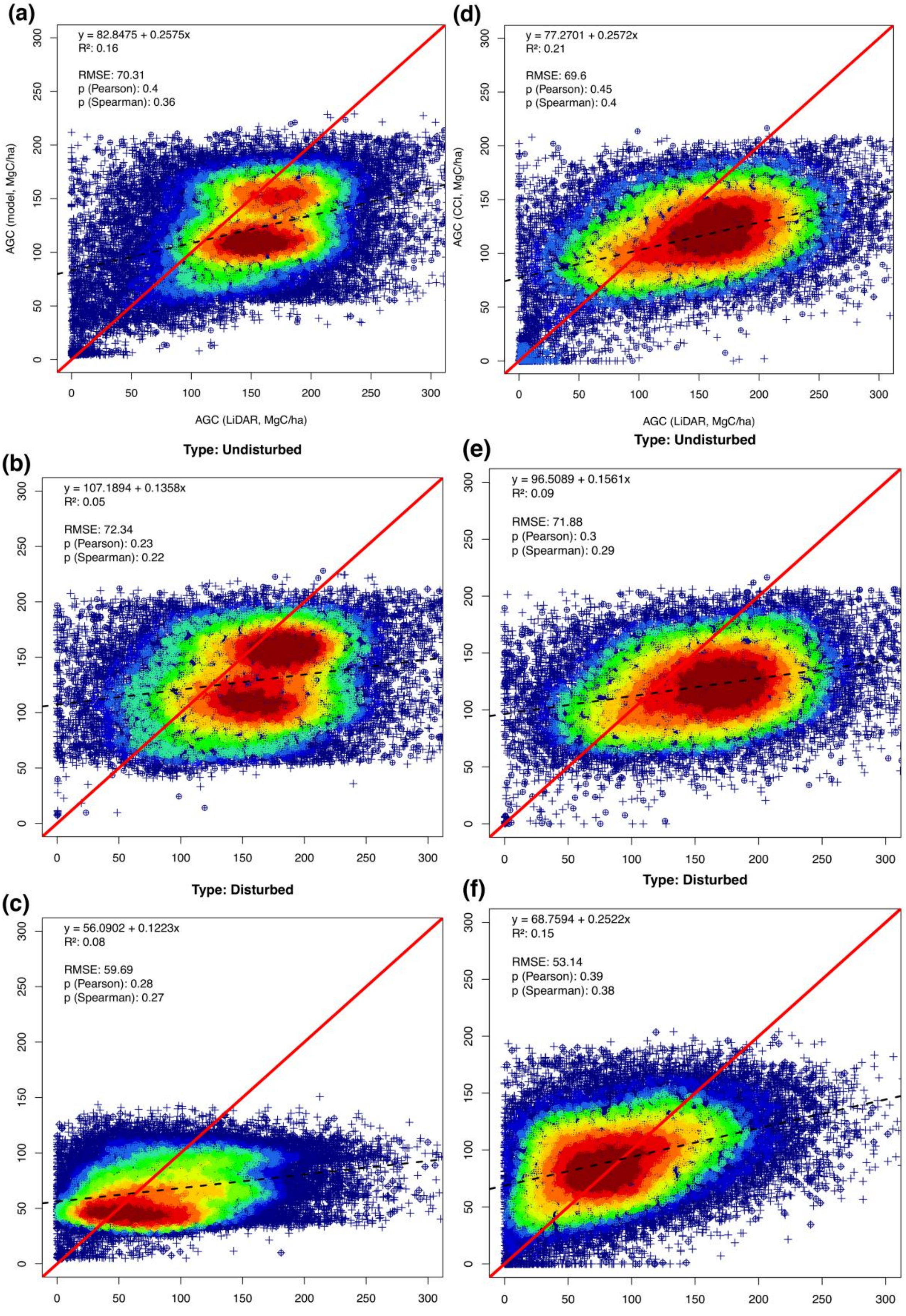

**Fig.S14. Assessment of AGC with LiDAR data (50m).** The plots compare the results of our model (left) and of the CCI dataset (right) with AGC estimates at 50m spatial resolution from Ometto et al. (2023). Each row details the comparison based either on deforested (a,d), regrowth (b,e) or other cover (c,f) pixels.

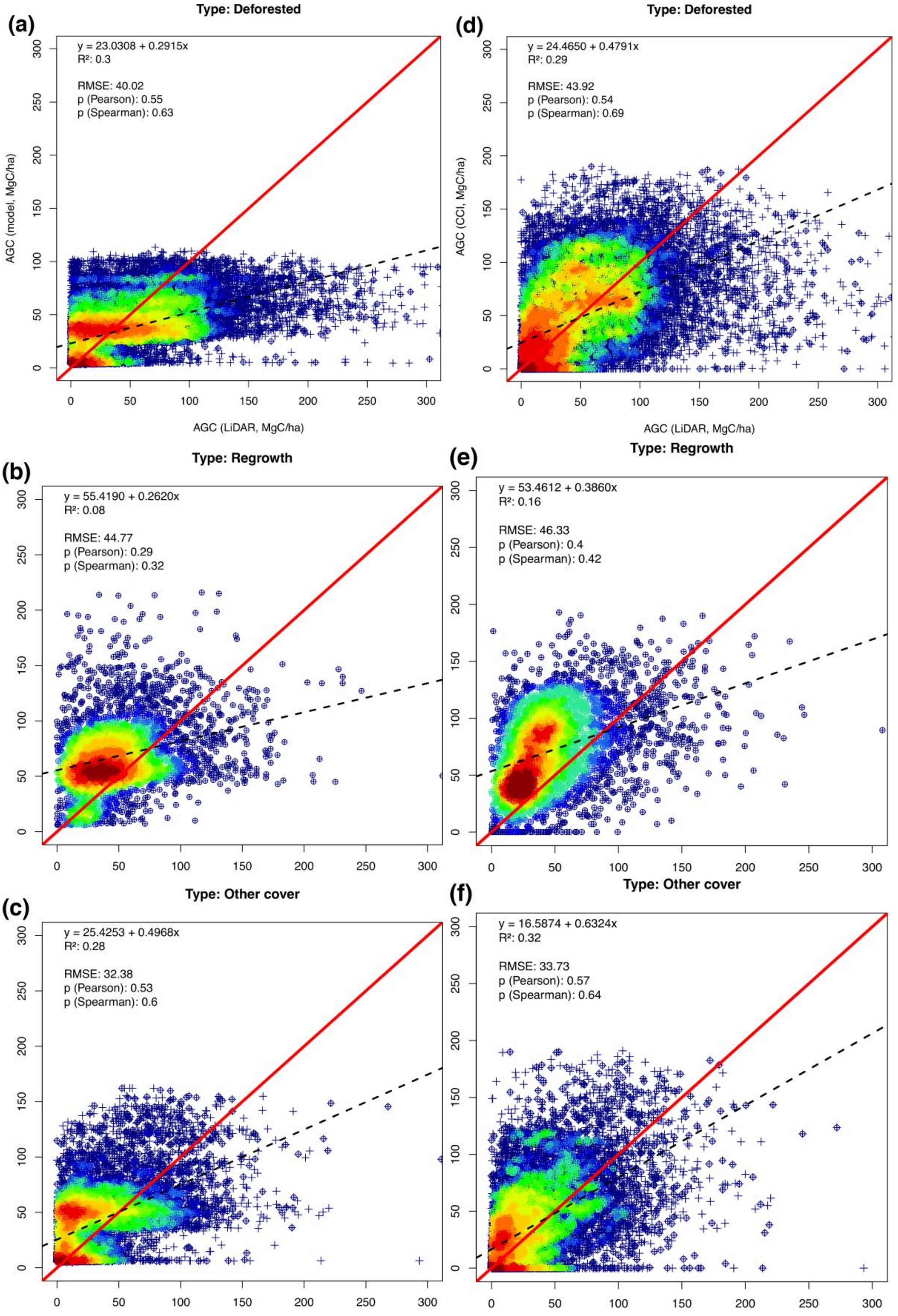

**Table S2. Summary of the validation of our model and CCI against the pixels of Ometto et al. (2023) with LiDAR information, at a 50m spatial resolution.** To facilitate comparison, the best values are highlighted in green.

| Pixels considered | Root mean square error (RMSE) | Pearson correlation coefficient | Spearman correlation coefficient | Coefficient of determination ($R^2$) |
|---|---|---|---|---|
| All pixels | Our work: 70.31<br>**CCI: 69.60 (-1.0%)** | Our: 0.40<br>**CCI: 0.45** | Our: 0.36<br>**CCI: 0.40** | Our: 0.16<br>**CCI: 0.21** |
| Undisturbed only | Our work: 72.34<br>**CCI: 71.88 (-0.6%)** | Our: 0.23<br>**CCI: 0.30** | Our: 0.22<br>**CCI: 0.29** | Our: 0.05<br>**CCI: 0.09** |
| Disturbed only | Our work: 59.69<br>**CCI: 53.14 (-11.0%)** | Our: 0.28<br>**CCI: 0.39** | Our: 0.27<br>**CCI: 0.37** | Our: 0.08<br>**CCI: 0.15** |
| Deforested only | **Our work: 40.02 (-8.9%)**<br>CCI: 43.92 | **Our: 0.55**<br>CCI: 0.54 | Our: 0.63<br>**CCI: 0.69** | **Our: 0.30**<br>CCI: 0.29 |
| Regrowth only | **Our work: 44.77 (-3.4%)**<br>CCI: 46.33 | Our: 0.29<br>**CCI: 0.40** | Our: 0.32<br>**CCI: 0.42** | Our: 0.08<br>**CCI: 0.16** |
| Other cover only | **Our work: 32.38 (-4.0%)**<br>CCI: 33.73 | Our: 0.53<br>**CCI: 0.57** | Our: 0.60<br>**CCI: 0.64** | Our: 0.28<br>**CCI: 0.32** |

The results do not depart much from the analysis at a 250m spatial resolution, with CCI having a stronger relationship in most cases, except the Pearson correlation and the coefficient of determination for deforested areas. A direct comparison of values indicates an RMSE 1.0% lower for CCI when all pixels are pooled together or 0.6% and 11.0% lower when only undisturbed or disturbed forests are considered, respectively. For deforested, regrowth, and other cover pixels, our model presented RMSEs lower by 8.9%, 3.4%, 28.1%, and 4.0%, respectively.

## C6. Limitations

The model builds on two independent datasets to reconstruct large scale gradients in forest biomass while preserving fine grain detail and consistent temporal trends. To do this, the model attempts to seek an agreement between the two input data streams by reconciling noisy observations and potentially diverging trends. On occasions, the model returns AGC values that, compared to available products, (i) tend to underestimate the effect of deforestation on AGC and (ii) may result in the death of trees followed by a second increase in AGC after forest regrowth (Fig.S15, top). Both effects seemed persistent even after many modifications and tests with the disaggregation model, and can be seen in Fig.S15a-b and S15c.

**Fig.S15. Distribution of the disaggregated AGC stocks (~ 100m spatial resolution) and fluxes per land cover class.** a-d: AGC stocks in 2020 per land cover class, considering all pixels (**a**), AGC stocks for pixels with land use change between 2010 and 2020 (**b**), delta AGC in 2010-2020 for pixels that remained unchanged during the period (**c**), delta AGC in 2010-2020 for pixels that changed during the period (**d**). The distributions result from a sample of size 1 million taken from the high-resolution data. Values above the violin plot correspond to the average stock or flux, and the share, in area, of the pixels within that category.

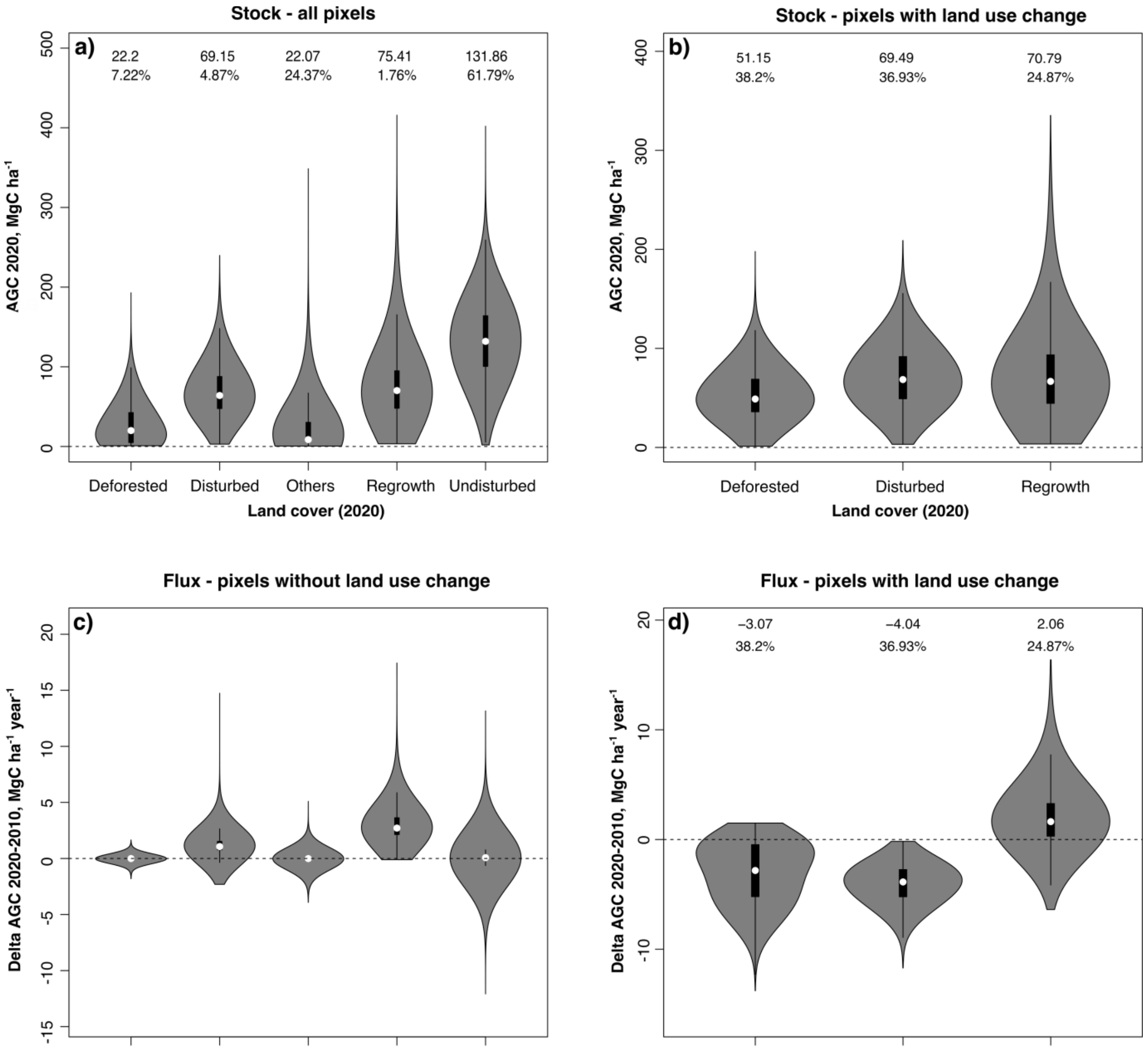


A first factor contributing to pattern (i) above is the lagged effect of deforestation on standing biomass. The complete removal of woody material after deforestation may take a few years. In the typical slash-and-burn process, a long delay may exist between when the forest cut down is burned, and the fire may not be able to combust all biomass at once, leaving residues on the ground. In both cases, the process could leave residues that affect the signal captured by the L-band. Besides, the land cover dataset used (i.e., TMF [12]) may mark as "deforestation" a pixel where deforestation was followed by agricultural expansion, and a comparison in the Brazilian Amazon (see "*Effect of the land cover change dataset*" next) shows that newly deforested areas in TMF exceed INPE's PRODES [14] deforestation estimates during all years in 2010-2020. In 2014, for example, the estimates of TMF were nearly four times higher than PRODES', which may have induced an underestimation of the effect of deforestation in the disaggregation model.

An alternative and empirical explanation for the pattern is given by the VOD data itself. Each VOD cell contains a share of fine-scale pixels with one of the land cover classes described in the Main Text. If VOD cells are split according to the percentiles of the share of deforestation pixels, the top 11.1% cells with the highest deforestation (i.e., denoted Q8 in Fig.S16) have a median stock always between 25 and 50 MgC/ha, relatively large compared to the 0 MgC/ha expected. This range is similar to the median 49.6 MgC/ha found in disaggregated pixels where deforestation happened between 2010 and 2020, indicating that the model ended up underestimating the impact of such class of land cover change. It is also noteworthy that, despite such limitations, our product still had a lower RMSE when compared to LiDAR data than ESA CCI in the Brazilian Amazon (Table S1, Table S2).

**Fig.S16. Boxplot of the VOD AGC data split according to percentiles of the share of deforestation, for all years.** In the horizontal axis, the notations Q1 and Q8 refer to the lowest and highest one-eight fractions of undisturbed forests per 0.25° cell, respectively.

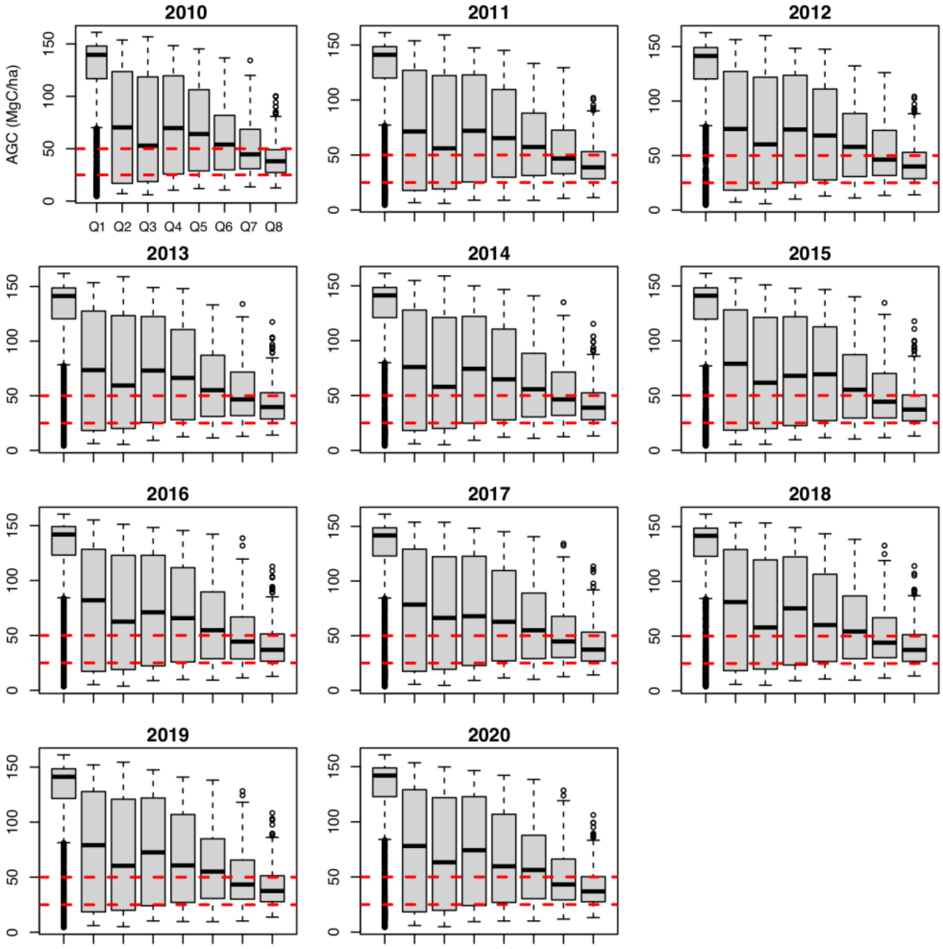


In the case of forest recovery paths (i.e., the pattern ii above), both successional dynamics and climatic effects provide feasible explanations, besides the interaction with the loss of remnant biomass and potential issues arising from misclassified pixel locations. In general, the closure of the canopy in a recovering forest path may result in an AGC decrease due to plant competition and self-thinning, as well as changes in species composition. At the same time, secondary forests have lower wood density and are more vulnerable to droughts. During the study period, the Amazon experienced important droughts, especially in 2015/2016, which may have contributed to reducing the biomass of recovering forest patches. The fact that a similar decay in the regrowth pattern was also observed in the coarse VOD data supports the second explanation. As seen in Fig.S17, median VOD AGC stocks always decrease with an increase in regrowth fraction, contrary to

expectation. This result suggests a potential confounding in the original data. In the disaggregated dataset, the small fraction of pixels marked as "forest regrowth" for the whole period of study (i.e., < 1%) end up having a median AGC increase of 3.0 MgC/ha.

**Fig.S17. Boxplot of the VOD AGC data split according to percentiles of the share of regrowth forests, for all years.** The notations Q1 and Q8 refer to the lowest and highest one-eight fractions of regrowth forests per 0.25° cell, respectively.

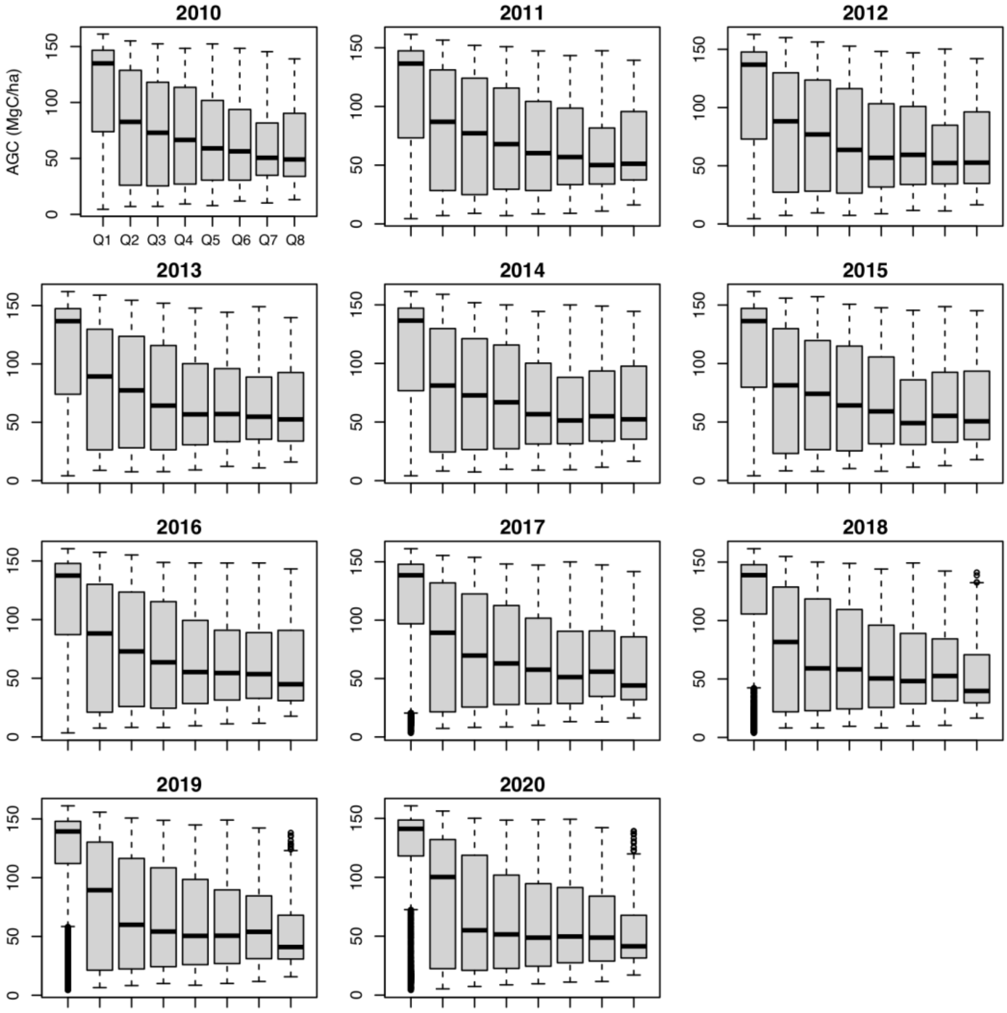


***Effect of the land cover change input dataset***

One potential source of uncertainty for the patterns observed for deforestation and regrowth could be the adoption of the TMF [12] dataset, indicating that a comparison against other sources of information could provide insights into intrinsic biases. To include the most datasets possible, such a comparison was calculated for the Brazilian Amazon. For deforestation (Fig.S18, top), newly deforested areas in TMF were generally higher than Mapbiomas' (primary forest only) [13] and INPE's PRODES estimates, except in the last three years, when TMF and Mapbiomas (primary forest only) converge. In 2014, TMF estimates are nearly four times higher than INPE's and twice those of Mapbiomas (primary forest only). From 2016 to 2018, the Global Forest Change estimates exceed the values in all other datasets. The comparison of deforestation rates does not suggest a clear or evident bias in the statistical disaggregation model. However, part of the underestimation of the

effect of deforestation reported previously may be related to an overestimation of this land cover class in TMF from 2010 to 2015.

Fewer datasets are available for comparison for regrowth (Fig.S18, bottom). Compared to Mapbiomas, TMF shows lower rates in the first half of the decade and the opposite pattern in the second half. The time series from the two datasets do not follow a similar trend over time, with the TMF dataset indicating an increase in forest regrowth over time. Such an increase may result from the misclassification of pixels where regrowth did not happen, which would partially explain the estimated effect trends from the fifth year onwards (Fig.S18, bottom right).

**Fig.S18. Rates of deforestation (top) and regrowth (bottom) in the Brazilian Amazon, across datasets.**

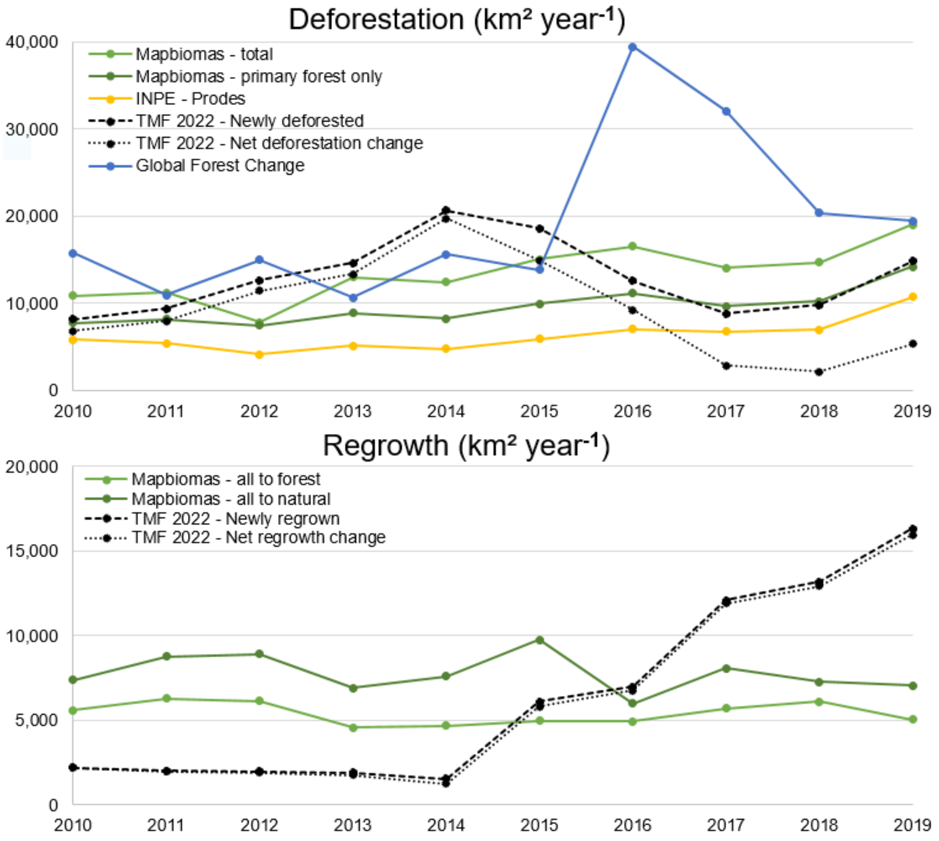


The overall distribution of land use classes in the TMF dataset within the study area may also be important to understand the results of the current work. Fig.S19 displays such information averaged over 2010-2020.

**Fig.S19. Area per land use class averaged over 2010-2020, according to the TMF dataset.**

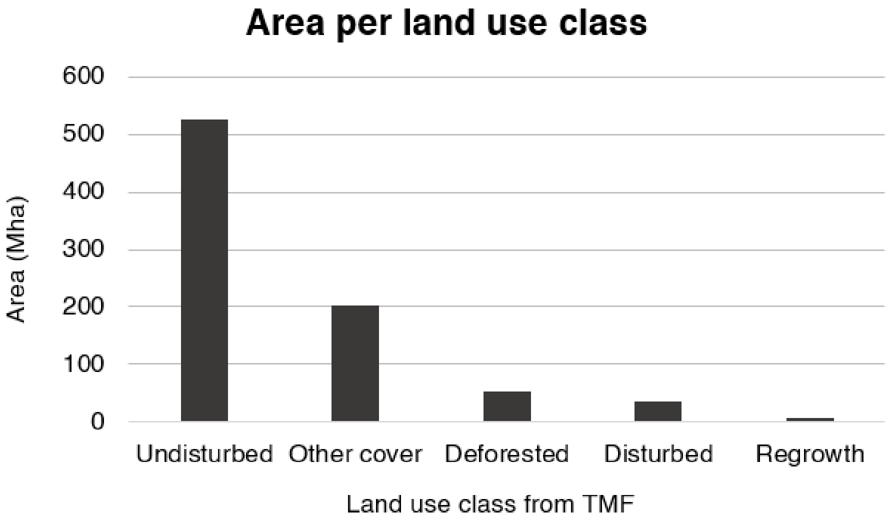


### *Effect of the AGC input dataset*

Another potential explanation for the lower deforestation effect could be an adjacency effect. In that case, the model could be obtaining low AGC at the coarse level not by averaging low AGC in deforested areas with high AGC in undisturbed forests (e.g., 50 as the average of 0 and 100 MgC ha$^{-1}$), but by assigning higher and lower AGC values to deforested and undisturbed forests, respectively (e.g., 50 as the average of 40 and 60 MgC ha$^{-1}$). In that case, the loss of such forests would be small in magnitude.

A plot of the average AGC in undisturbed forests against their fraction at the coarse level helps investigate this issue. Using the TMF dataset for the undisturbed forests, such a plot was generated for three high-resolution maps: our model and CCI [6] for the whole Amazon, and Ometto et al. (2023) [17] for the Brazilian Amazon. As shown in Fig.S20, while the model indeed assigns lower AGC for cells with a lower fraction of undisturbed forests, such a pattern is also found in other published products, indicating that it is more likely to be due to different factors, such as the presence of border effects, than some particular model misbehavior.

**Fig.S20. Comparison of the relationship between the average AGC in undisturbed forests and their fraction in three different products.**

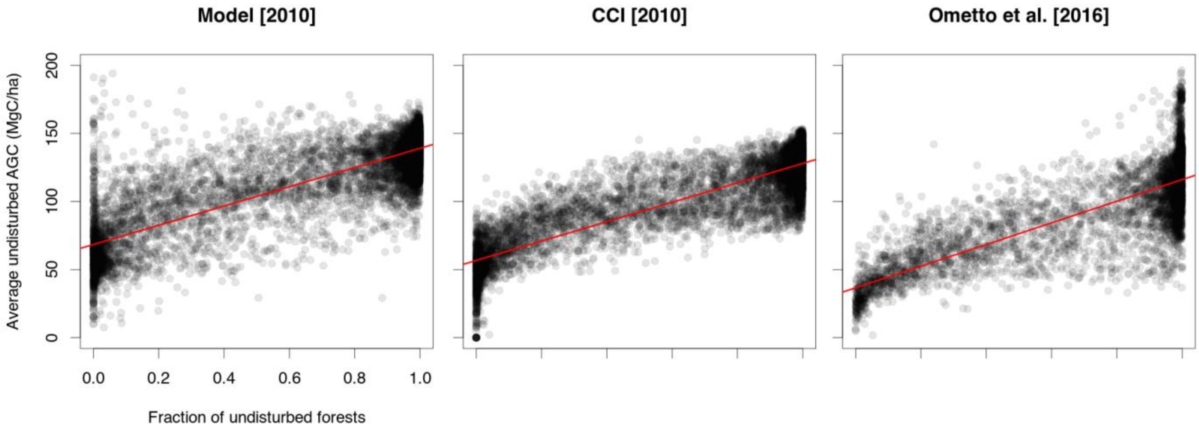

***Effect of forcing model behavior***

The standard implementation of the disaggregation model described so far estimates the relationships from the data, considering all assumptions presented in Section C1. However, model identification for some patterns can be challenging due to interactions, nonlinear effects, and other reasons [15]. In that case, techniques such as adopting constrained shape-constrained splines can help by imposing restrictions on the behavior of the estimated patterns. Such a technique could, for example, force the trajectory after regrowth to be a monotonic non-decreasing function. However, imposing shape constraints on the splines used in the current work requires re-parameterizing them and modifying the estimation procedure [16], two things that cannot be easily done within the current implementation of the disaggregation model. Yet, adding the extra assumption of forcing deforested pixels to have zero AGC remains possible, and this exercise was developed to understand what would happen with the disaggregation model in that case.

The results indicate that forcing deforestation to have zero AGC would lead to worse model behavior. First, the Akaike Information Criterion would increase, with the difference being $\Delta AIC = 1580.6$. Such a change indicates an inferior tradeoff between model fit and complexity when the model is forced. The mean squared error between predicted AGC and VOD data at the coarse level would also increase from 12.996 MgC ha$^{-1}$ in the current model to 13.216 MgC ha$^{-1}$ in the forced model. Besides, forcing the deforestation behavior would lead to a loss in the currently reasonable ordering estimated for the land cover type effect, with the new results indicating a small increase in AGC after a transition of a pixel from an undisturbed to a disturbed state (Fig.S21, top). The effect of regrowth over time, although currently non-intuitive (see *"C6. Limitations"*), would become decreasing and statistically non-significant (Fig.S21, bottom), indicating a behavior of negligible decrease of AGC after regrowth.

**Fig.S21. Comparison of the smoothers obtained in the current and the forced model**

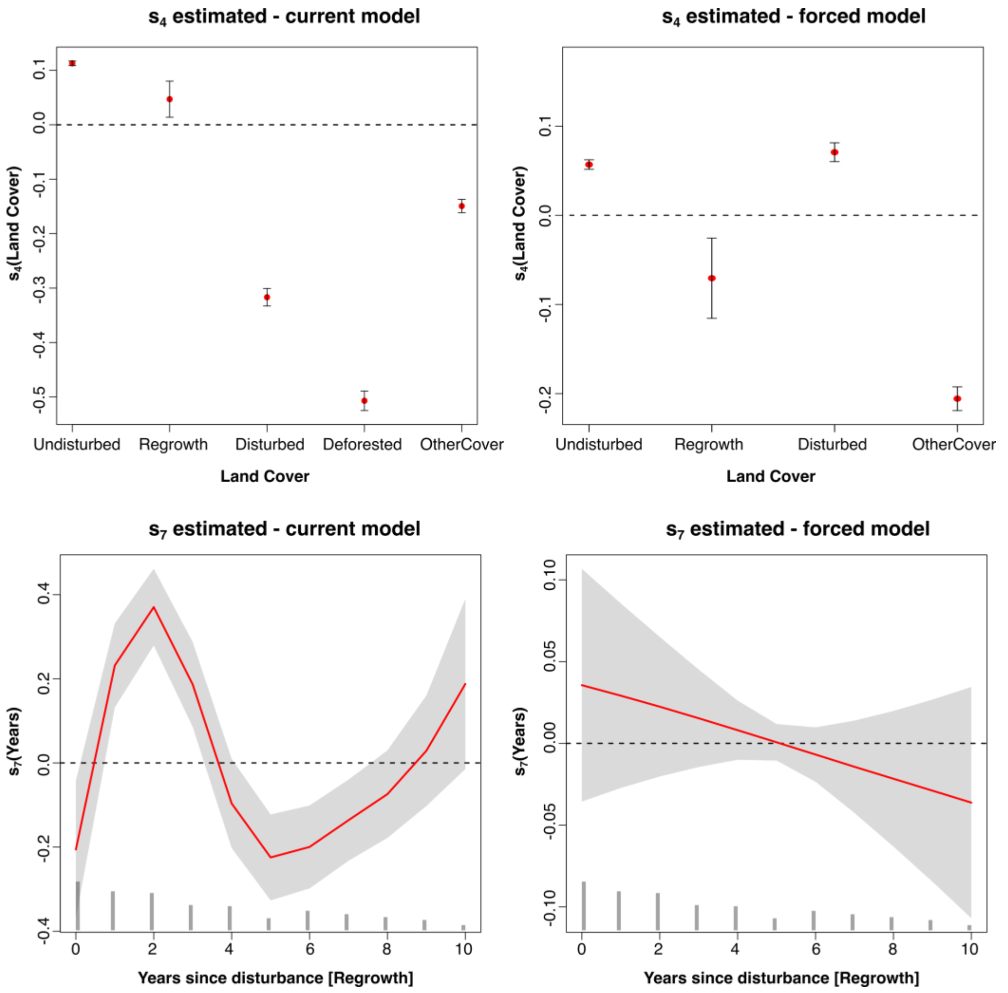

## C7. Other relevant information

**Fig.S22. Schematic representation of the methodology adopted.**

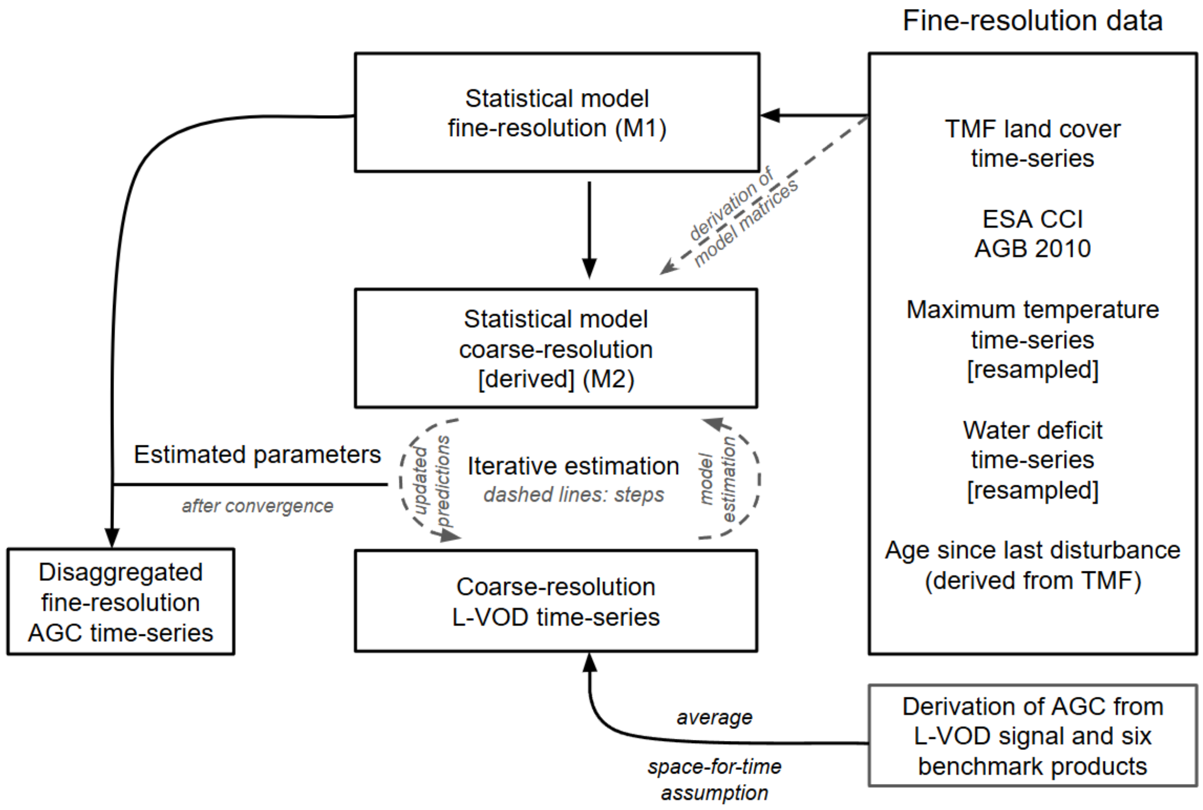


**Fig.S23. Location of the study areas mentioned in the manuscript.** The boundary referred to as "Peruvian Amazon" correspond to that used by Asner et al. (2010) [18].

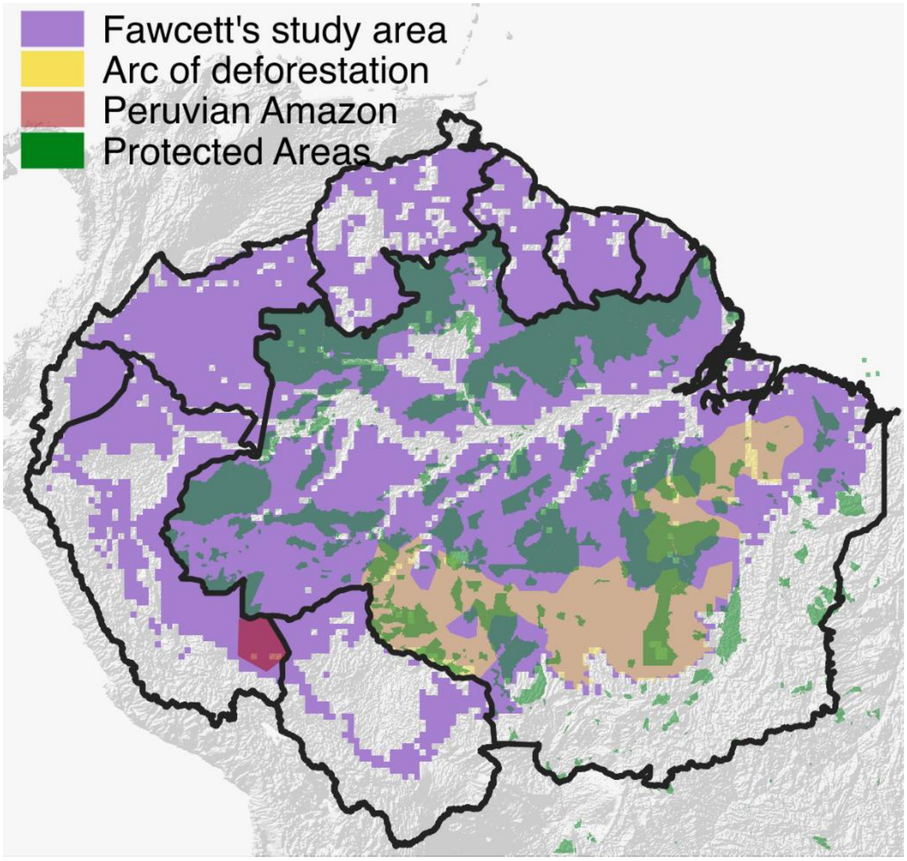

**Fig.S24. Share of gross AGC gains (top) and losses (bottom) in 2019 in each land cover class.**

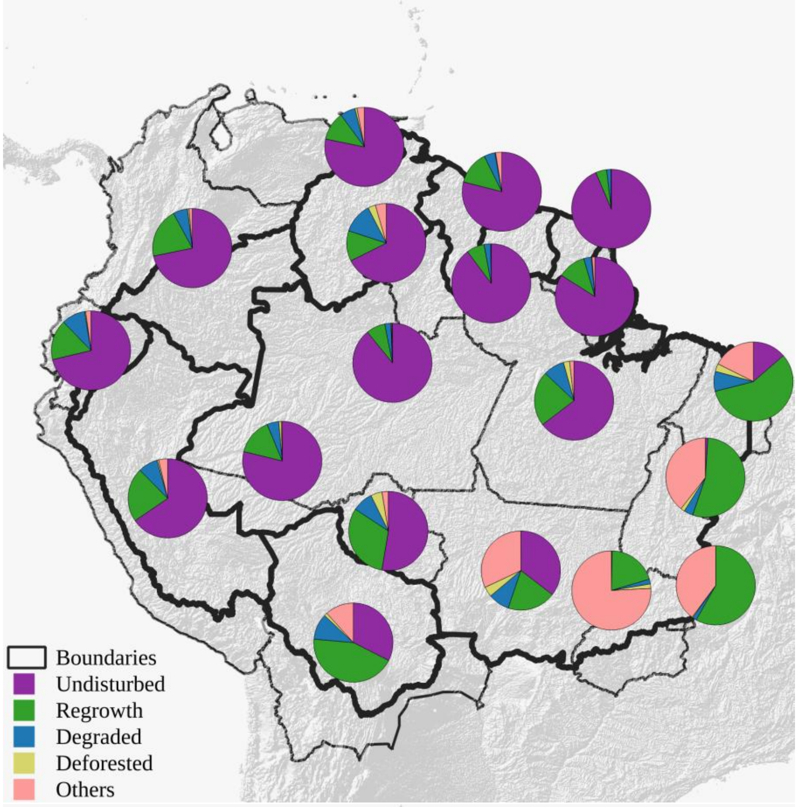


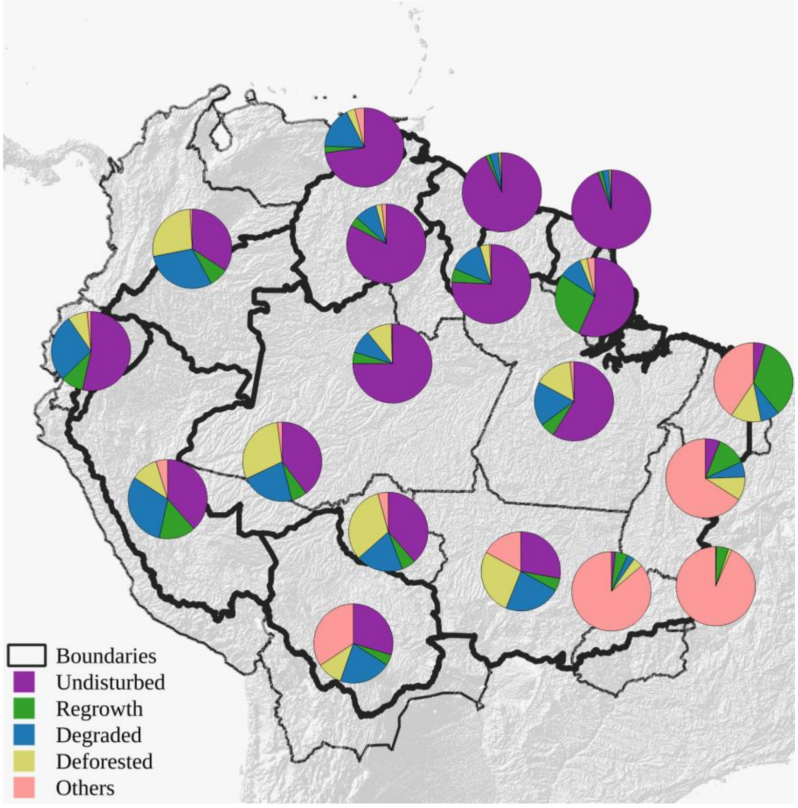

**Table S3. Gross annual AGC change over 2010-2020 and area per land cover class for the whole simulation domain.** The rows in each cell correspond to: 1) gross annual AGC gain (MgC $ha^{-1}$ $yr^{-1}$); 2) area with AGC gain (ha); 3) gross annual AGC loss (MgC $ha^{-1}$ $yr^{-1}$); 4) area with AGC loss (ha). The product between rows 1 and 2 [or rows 3 and 4] yields the average gains [or losses] of Fig. 2c-d, and the total (i.e., sum) leads to average net annual values of Fig. 2b. The columns “Newly Deforested” and “Previously Deforested” are subcategories of “Deforested [average]” and are not be included in the “Total change”.

| Year | Undisturbed | Regrowth | Degraded | Deforested [average] | Previously Deforested | Newly Deforested | Other cover | Total change |
|---|---|---|---|---|---|---|---|---|
| 2010 | 1.76 MgC $ha^{-1}$ $yr^{-1}$<br>4.72 $10^8$ ha<br>-1.27 MgC $ha^{-1}$ $yr^{-1}$<br>0.64 $10^8$ ha | 32.42<br>0.04 $10^8$<br>-0.73<br>0.00 $10^8$ | 1.49<br>0.27 $10^8$<br>-14.24<br>0.05 $10^8$ | 0.42<br>0.26 $10^8$<br>-1.75<br>0.21 $10^8$ | 0.42<br>0.26 $10^8$<br>-0.30<br>0.19 $10^8$ | 0.66<br>0.00 $10^8$<br>-22.90<br>0.01 $10^8$ | 0.54<br>1.21 $10^8$<br>-0.47<br>0.84 $10^8$ | 1.64<br>6.49 $10^8$<br>-1.28<br>1.73 $10^8$ |
| 2011 | 0.93<br>3.59 $10^8$<br>-1.32<br>1.76 $10^8$ | 15.84<br>0.04 $10^8$<br>-0.86<br>0.00 $10^8$ | 1.28<br>0.26 $10^8$<br>-6.66<br>0.06 $10^8$ | 0.30<br>0.19 $10^8$<br>-1.44<br>0.29 $10^8$ | 0.30<br>0.19 $10^8$<br>-0.32<br>0.27 $10^8$ | 0.26<br>0.00 $10^8$<br>-23.15<br>0.01 $10^8$ | 0.48<br>0.93 $10^8$<br>-0.50<br>1.11 $10^8$ | 0.96<br>5.01 $10^8$<br>-1.15<br>3.22 $10^8$ |
| 2012 | 1.26<br>1.42 $10^8$<br>-1.16<br>3.91 $10^8$ | 18.91<br>0.01 $10^8$<br>-17.86<br>0.03 $10^8$ | 1.39<br>0.28 $10^8$<br>-13.88<br>0.04 $10^8$ | 0.27<br>0.27 $10^8$<br>-2.27<br>0.23 $10^8$ | 0.27<br>0.27 $10^8$<br>-0.25<br>0.21 $10^8$ | 3.81<br>0.00 $10^8$<br>-23.58<br>0.02 $10^8$ | 0.38<br>0.96 $10^8$<br>-0.47<br>1.08 $10^8$ | 0.96<br>2.94 $10^8$<br>-1.27<br>5.29 $10^8$ |
| 2013 | 1.62<br>1.79 $10^8$<br>-1.45<br>3.52 $10^8$ | 19.28<br>0.01 $10^8$<br>-21.22<br>0.04 $10^8$ | 1.77<br>0.27 $10^8$<br>-10.86<br>0.04 $10^8$ | 0.48<br>0.32 $10^8$<br>-3.06<br>0.19 $10^8$ | 0.48<br>0.32 $10^8$<br>-0.33<br>0.17 $10^8$ | 0.73<br>0.00 $10^8$<br>-25.06<br>0.02 $10^8$ | 0.61<br>1.35 $10^8$<br>-0.50<br>0.68 $10^8$ | 1.22<br>3.75 $10^8$<br>-1.63<br>4.48 $10^8$ |
| 2014 | 1.07<br>2.73 $10^8$<br>-1.66<br>2.55 $10^8$ | 21.01<br>0.01 $10^8$<br>-10.72<br>0.04 $10^8$ | 1.46<br>0.24 $10^8$<br>-11.20<br>0.09 $10^8$ | 0.34<br>0.18 $10^8$<br>-2.53<br>0.36 $10^8$ | 0.34<br>0.18 $10^8$<br>-0.46<br>0.33 $10^8$ | 1.63<br>0.00 $10^8$<br>-27.84<br>0.03 $10^8$ | 0.48<br>0.87 $10^8$<br>-0.62<br>1.17 $10^8$ | 0.99<br>4.02 $10^8$<br>-1.74<br>4.20 $10^8$ |
| 2015 | 1.44<br>3.52 $10^8$<br>-1.52<br>1.71 $10^8$ | 6.87<br>0.04 $10^8$<br>-9.13<br>0.02 $10^8$ | 1.62<br>0.26 $10^8$<br>-20.54<br>0.08 $10^8$ | 0.34<br>0.23 $10^8$<br>-3.08<br>0.33 $10^8$ | 0.34<br>0.23 $10^8$<br>-0.44<br>0.30 $10^8$ | 2.12<br>0.00 $10^8$<br>-32.72<br>0.03 $10^8$ | 0.48<br>0.90 $10^8$<br>-0.48<br>1.13 $10^8$ | 1.27<br>4.96 $10^8$<br>-1.86<br>3.27 $10^8$ |
| 2016 | 1.03<br>2.41 $10^8$<br>-1.27<br>2.79 $10^8$ | 9.13<br>0.05 $10^8$<br>-10.23<br>0.02 $10^8$ | 1.41<br>0.28 $10^8$<br>-12.83<br>0.08 $10^8$ | 0.32<br>0.17 $10^8$<br>-2.10<br>0.40 $10^8$ | 0.32<br>0.17 $10^8$<br>-0.47<br>0.38 $10^8$ | 0.00<br>0.00 $10^8$<br>-34.61<br>0.02 $10^8$ | 0.44<br>0.75 $10^8$<br>-0.52<br>1.27 $10^8$ | 1.03<br>3.66 $10^8$<br>-1.38<br>4.56 $10^8$ |
| 2017 | 0.92<br>1.43 $10^8$<br>-1.18<br>3.74 $10^8$ | 10.66<br>0.07 $10^8$<br>-9.41<br>0.02 $10^8$ | 1.51<br>0.33 $10^8$<br>-17.98<br>0.05 $10^8$ | 0.29<br>0.29 $10^8$<br>-2.06<br>0.29 $10^8$ | 0.29<br>0.29 $10^8$<br>-0.33<br>0.28 $10^8$ | 0.14<br>0.00 $10^8$<br>-36.31<br>0.01 $10^8$ | 0.33<br>1.00 $10^8$<br>-0.36<br>1.01 $10^8$ | 0.96<br>3.12 $10^8$<br>-1.26<br>5.11 $10^8$ |
| 2018 | 1.51<br>2.18 $10^8$<br>-1.10<br>2.96 $10^8$ | 13.34<br>0.09 $10^8$<br>-10.58<br>0.02 $10^8$ | 1.78<br>0.32 $10^8$<br>-14.33<br>0.06 $10^8$ | 0.41<br>0.28 $10^8$<br>-2.08<br>0.30 $10^8$ | 0.41<br>0.28 $10^8$<br>-0.38<br>0.28 $10^8$ | 1.94<br>0.00 $10^8$<br>-34.95<br>0.01 $10^8$ | 0.40<br>1.02 $10^8$<br>-0.56<br>0.97 $10^8$ | 1.44<br>3.91 $10^8$<br>-1.29<br>4.32 $10^8$ |
| 2019 | 1.83<br>3.28 $10^8$<br>-1.78<br>1.83 $10^8$ | 15.14<br>0.11 $10^8$<br>-11.65<br>0.04 $10^8$ | 1.94<br>0.30 $10^8$<br>-12.54<br>0.10 $10^8$ | 0.51<br>0.26 $10^8$<br>-2.75<br>0.33 $10^8$ | 0.51<br>0.26 $10^8$<br>-0.57<br>0.31 $10^8$ | 0.87<br>0.00 $10^8$<br>-30.82<br>0.02 $10^8$ | 0.58<br>0.98 $10^8$<br>-0.81<br>1.00 $10^8$ | 1.82<br>4.93 $10^8$<br>-2.00<br>3.30 $10^8$ |

**Table S4. Gross annual AGC change over 2010-2020 and area per land cover class in Brazil, inside Protected Areas.** The rows in each cell correspond to: 1) gross annual AGC gain (TgC $yr^{-1}$); 2) area with AGC gain (ha); 3) gross annual AGC loss (TgC $yr^{-1}$); 4) area with AGC loss (ha).

| Year | Undisturbed | Regrowth | Degraded | Deforested [average] | Other cover |
|---|---|---|---|---|---|
| 2010 | 235.16 TgC $yr^{-1}$<br>119.98 Mha<br>-15.76 TgC $yr^{-1}$<br>13.55 Mha | 9.97<br>0.30<br>-0.01<br>0.01 | 3.61<br>2.29<br>-9.20<br>0.58 | 0.65<br>0.77<br>-3.95<br>0.95 | 4.54<br>8.59<br>-3.33<br>7.41 |
| 2011 | 85.16<br>88.30<br>-54.21<br>45.08 | 5.46<br>0.33<br>-0.01<br>0.01 | 3.23<br>2.36<br>-3.92<br>0.51 | 0.46<br>0.82<br>-3.46<br>1.02 | 3.17<br>8.08<br>-3.22<br>7.90 |
| 2012 | 45.04<br>38.60<br>-117.84<br>94.57 | 1.58<br>0.08<br>-5.29<br>0.28 | 3.24<br>2.49<br>-6.15<br>0.42 | 0.30<br>0.75<br>-4.90<br>1.25 | 2.65<br>6.63<br>-3.70<br>9.34 |
| 2013 | 80.26<br>49.72<br>-122.52<br>83.29 | 1.52<br>0.08<br>-7.00<br>0.32 | 4.22<br>2.46<br>-4.42<br>0.46 | 1.02<br>1.18<br>-3.93<br>0.95 | 5.48<br>8.77<br>-2.99<br>7.18 |
| 2014 | 71.96<br>63.78<br>-125.79<br>68.80 | 1.82<br>0.08<br>-3.78<br>0.34 | 3.02<br>2.10<br>-15.20<br>1.00 | 0.43<br>0.78<br>-8.22<br>1.59 | 2.93<br>7.20<br>-6.68<br>8.75 |
| 2015 | 130.96<br>81.51<br>-81.48<br>50.30 | 2.24<br>0.34<br>-1.34<br>0.14 | 4.01<br>2.43<br>-27.57<br>1.18 | 0.74<br>1.07<br>-9.33<br>1.53 | 4.23<br>7.43<br>-4.85<br>8.48 |
| 2016 | 63.00<br>60.73<br>-106.29<br>70.54 | 4.12<br>0.45<br>-1.49<br>0.14 | 4.27<br>2.91<br>-19.02<br>1.02 | 0.58<br>0.96<br>-8.76<br>1.82 | 3.01<br>6.19<br>-4.90<br>9.66 |
| 2017 | 39.86<br>38.11<br>-109.91<br>92.89 | 6.85<br>0.62<br>-1.50<br>0.15 | 5.84<br>3.54<br>-11.52<br>0.59 | 0.85<br>1.43<br>-3.62<br>1.34 | 2.82<br>8.82<br>-2.38<br>6.92 |
| 2018 | 90.32<br>59.05<br>-87.96<br>71.66 | 11.45<br>0.80<br>-1.95<br>0.18 | 5.81<br>3.41<br>-12.44<br>0.93 | 0.72<br>1.33<br>-4.04<br>1.40 | 3.57<br>8.41<br>-2.53<br>7.24 |
| 2019 | 162.95<br>80.16<br>-91.38<br>50.14 | 16.15<br>1.00<br>-3.44<br>0.30 | 6.75<br>3.39<br>-14.93<br>1.13 | 0.99<br>1.28<br>-8.41<br>1.55 | 4.09<br>7.04<br>-5.32<br>8.44 |

**Table S5. Gross annual AGC change over 2010-2020 and area per land cover class in Brazil, outside Protected Areas.** The rows in each cell correspond to: 1) gross annual AGC gain (TgC yr$^{-1}$); 2) area with AGC gain (ha); 3) gross annual AGC loss (TgC yr$^{-1}$); 4) area with AGC loss (ha).

| Year | Undisturbed | Regrowth | Degraded | Deforested [average] | Other cover |
|---|---|---|---|---|---|
| 2010 | 271.39 TgC yr$^{-1}$<br>152.06 Mha<br>-36.82 TgC yr$^{-1}$<br>29.26 Mha | 63.70<br>2.11<br>-0.04<br>0.05 | 20.85<br>13.41<br>-26.16<br>2.43 | 8.96<br>21.74<br>-20.60<br>15.63 | 42.04<br>65.70<br>-25.34<br>50.06 |
| 2011 | 119.58<br>118.48<br>-78.45<br>62.05 | 32.43<br>2.29<br>-0.05<br>0.05 | 14.02<br>11.88<br>-16.64<br>3.83 | 4.10<br>15.00<br>-27.23<br>23.71 | 26.43<br>47.24<br>-34.75<br>68.45 |
| 2012 | 76.09<br>58.52<br>-146.04<br>120.89 | 9.46<br>0.55<br>-32.53<br>1.97 | 19.25<br>13.36<br>-22.25<br>2.21 | 5.81<br>22.62<br>-32.95<br>16.71 | 17.96<br>47.16<br>-33.32<br>68.44 |
| 2013 | 105.29<br>62.50<br>-194.55<br>115.81 | 9.23<br>0.54<br>-42.67<br>2.16 | 22.50<br>12.96<br>-20.56<br>2.33 | 11.18<br>25.97<br>-40.15<br>14.66 | 45.80<br>80.00<br>-15.34<br>35.51 |
| 2014 | 101.30<br>79.49<br>-159.12<br>96.71 | 8.46<br>0.49<br>-23.18<br>2.33 | 12.78<br>10.03<br>-52.95<br>5.42 | 3.84<br>12.83<br>-67.42<br>29.69 | 26.76<br>45.68<br>-43.46<br>69.76 |
| 2015 | 163.02<br>112.54<br>-102.17<br>60.29 | 12.94<br>2.23<br>-8.50<br>1.15 | 17.45<br>11.56<br>-107.29<br>5.50 | 5.59<br>18.30<br>-72.30<br>25.71 | 28.40<br>55.12<br>-31.05<br>60.03 |
| 2016 | 81.93<br>76.74<br>-149.76<br>94.23 | 26.51<br>3.07<br>-8.43<br>0.97 | 14.94<br>12.02<br>-50.96<br>5.70 | 3.55<br>12.21<br>-57.64<br>32.74 | 17.95<br>37.20<br>-43.73<br>77.54 |
| 2017 | 47.28<br>51.99<br>-130.96<br>117.57 | 40.77<br>4.32<br>-7.43<br>1.08 | 21.92<br>15.42<br>-39.91<br>2.87 | 6.00<br>23.00<br>-40.19<br>22.26 | 17.58<br>54.01<br>-19.88<br>59.92 |
| 2018 | 131.38<br>81.83<br>-105.80<br>86.35 | 69.11<br>5.42<br>-12.14<br>1.23 | 28.47<br>15.06<br>-38.54<br>3.67 | 8.95<br>22.97<br>-41.84<br>22.60 | 22.76<br>58.74<br>-21.56<br>54.57 |
| 2019 | 239.73<br>107.78<br>-108.36<br>58.61 | 89.88<br>6.48<br>-21.24<br>1.88 | 30.71<br>14.48<br>-46.77<br>4.59 | 10.33<br>20.92<br>-56.75<br>25.25 | 41.16<br>56.28<br>-26.04<br>56.16 |

**Table S6. Statistics within Fawcett's study domain (Fig.S23).**

| Quantity | Value |
|---|---|
| Average 2020 AGC: deforested (MgC ha$^{-1}$) | 26.07 |
| Average 2020 AGC: disturbed (MgC ha$^{-1}$) | 70.81 |
| Average 2020 AGC: other areas (MgC ha$^{-1}$) | 21.05 |
| Average 2020 AGC: regrowth (MgC ha$^{-1}$) | 78.36 |
| Average 2020 AGC: undisturbed (MgC ha$^{-1}$) | 134.23 |
| Average newly-deforested rate 2010-2020 (km$^2$) | 14,595 |

**Fig.S25. Net annual AGC change per land cover category, within Fawcett's study domain (Fig.S23).**

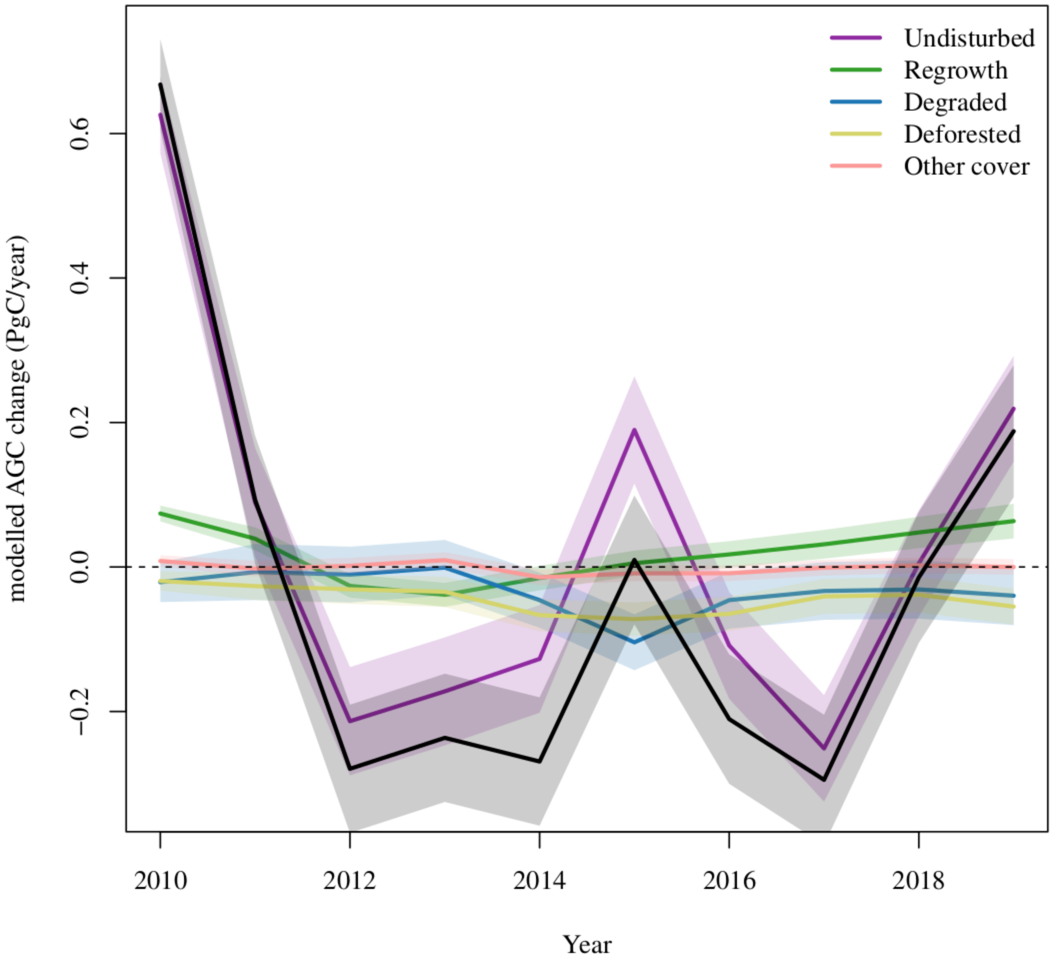

### *C7.1. Analysis per region*

Fig.3 of the Main Text shows an aggregation of the data per political boundaries. These were taken as the boundaries of federative units for Brazil, given its large size compared to the others, and the countries' boundaries elsewhere. Fig.S26 displays such boundaries along with the corresponding fractions of human-influenced land.

**Fig.S26. Fraction of human-influenced land within Amazonian boundaries per political region.**

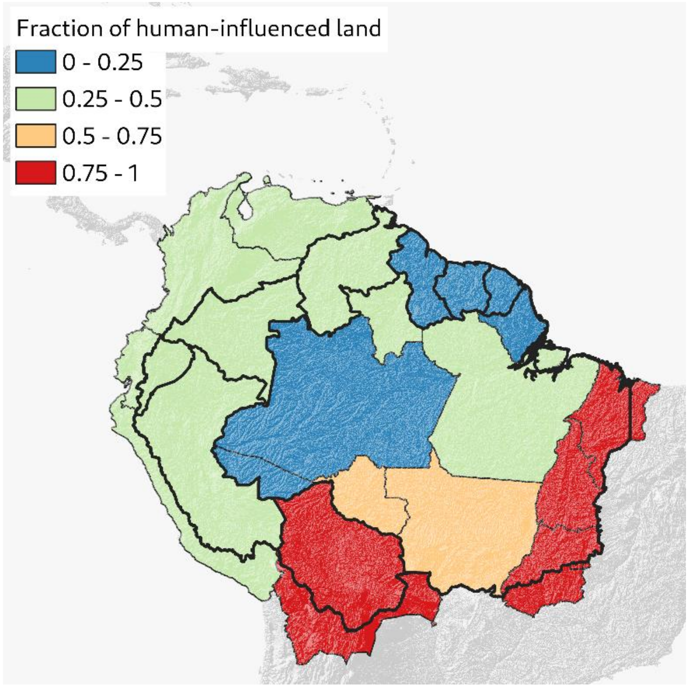


To test for the unit slope in the relationship between the fraction of the total area with gains [or losses] (below referred to as *y*) that is human-influenced and the fraction of the total AGC gains [or losses] (below referred to as *x*) that they represent, a linear regression model was used, $y = b_o + b_1 x + \epsilon$, where $b_o$ is the intercept, $b_1$ is the slope, and $\epsilon$ is the independent and identically distributed Gaussian errors. Then, a hypothesis test was set up, with a null hypothesis ($H_0$) indicating unit slope, and an alternative hypothesis ($H_1$) indicating otherwise:

$$H_0 :\ b_1 = 1$$

$$H_1 :\ b_1 \neq\ 1$$

The result of the hypothesis test for the losses (i.e., left of Fig.3c and Fig.3d) and the gains (i.e., right of Fig.3c and Fig.3d) are shown in Table S7, followed by a graphical representation in Fig.S27. The results show that $H_0$ can not be rejected for the gains, but it can be rejected at the 0.05 significance level for the losses.

**Table S7. Results of the hypothesis test for unit slope in the linear regression model.**

| Type | Linear regression | | Hypothesis test | |
|---|---|---|---|---|
| | $b_1$ | Standard error | F-statistic | p-value |
| Gains | 1.06 | 0.051 | 1.436 | 0.2472 |
| Losses | 1.20 | 0.089 | 5.014 | 0.0388 |

**Fig.S27. Graphical visualization of the unit-slope test.**

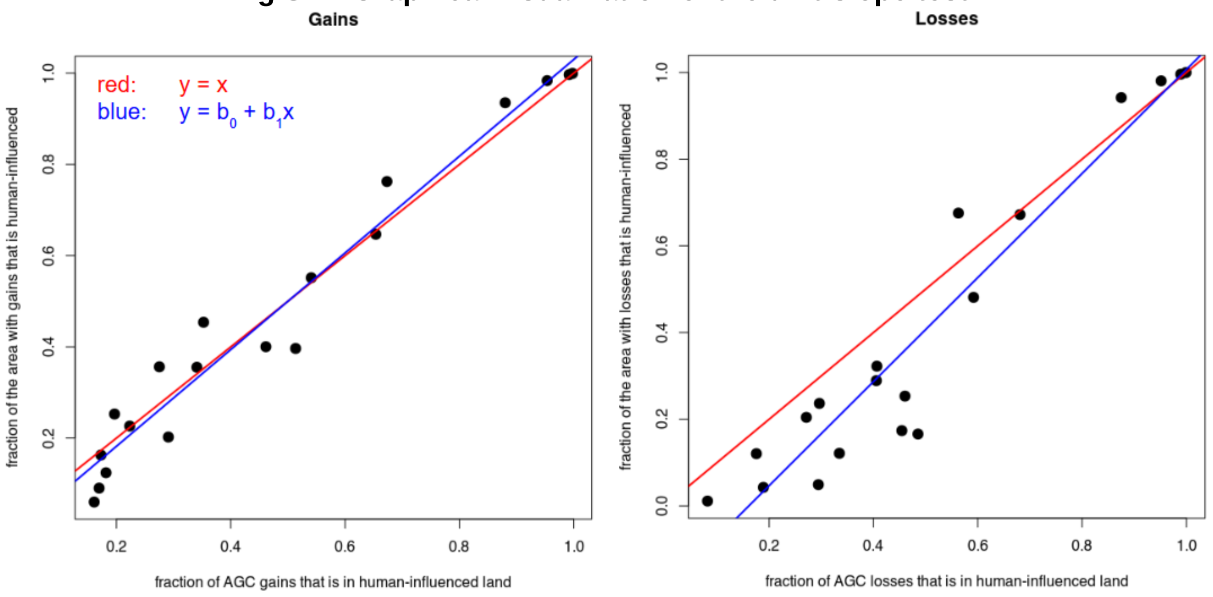


Analogously, a hypothesis test was also set up to check if the share of AGC losses in human-influenced land increased at a faster pace than the share of the total area with losses that were human-influenced land from the first to the second half of the last decade. In that case, a one-sample paired t-test with the following hypotheses was performed:

$$H_0 : \mu_{AGC} - \mu_{AREA} = 0$$

$$H_1 : \mu_{AGC} - \mu_{AREA} > 0$$

Where $\mu_{AGC}$ is the mean variation in the share of AGC losses in human-influenced land (Fig.3e, left), and $\mu_{AREA}$ is the mean variation of the share of the total area with losses that were human-influenced land (Fig.3e, right). The results of Table S8 show that $H_0$ can be rejected at the 0.01 significance level.

**Table S8. Results of the hypothesis test for Fig. 3e.**

| Type | Hypothesis test | | | |
|---|---|---|---|---|
| | $\mu_{AGC} - \mu_{AREA}$ | T-statistic | Degrees of freedom | p-value |
| Gains | 0.0360 | 2.5564 | 18 | 0.0099 |

### *C7.2. Analysis of the intensity of losses in human-influenced land*

A hypothesis test was performed to evaluate if the mean intensity of losses remained the same from the first half to the second half of the decade. Because the average intensities calculated using our maps for the first (i.e., -1.57 MgC $ha^{-1}$) and second (i.e., -1.85 MgC $ha^{-1}$) half of the decade do not have an associated standard deviation, we adopted a bootstrapping approach. This was done by repeatedly recalculating the average from a sample of 5,000 points randomly distributed across the Amazon. A total of 250 repetitions were performed, resulting in the intensities of Fig.S27.

**Fig.S28. Intensity of AGC losses in human-influenced land, per year.**

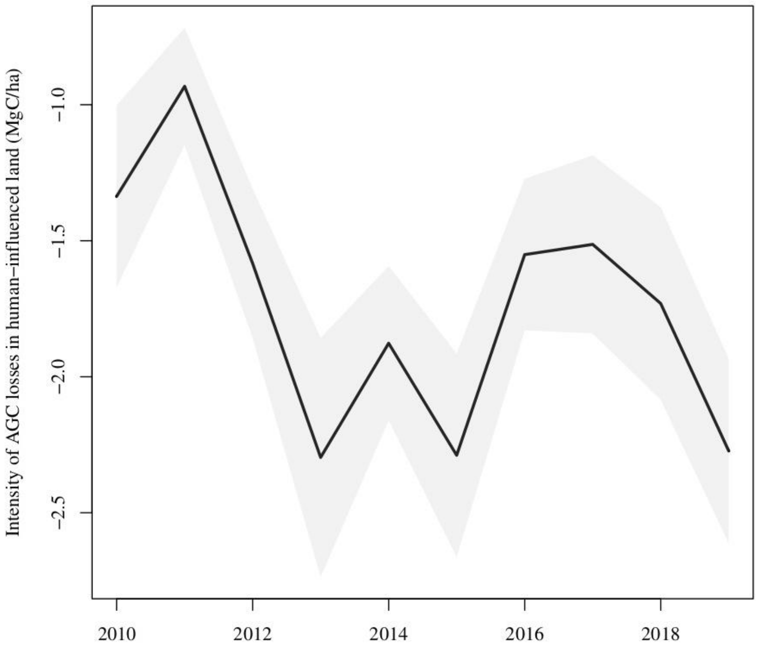


The two-sample paired t-test was performed to assess whether the mean intensity in the first half of the decade is equal to that of the second half ($H_0$), with an alternative hypothesis ($H_1$) indicating otherwise:

$$H_0 : \mu_{P1} = \mu_{P2}$$

$$H_1 : \mu_{P1} \neq \mu_{P2}$$

Where $\mu_{P1}$ is the mean intensity in the first period and $\mu_{P2}$, in the second period. The results shown in Table S9 indicate that $H_0$ can be rejected at the 0.001 significance level.

**Table S9. Results of the two-sample paired t-test for the intensity of losses.**

| Hypothesis test | | | | |
|---|---|---|---|---|
| $\mu_{P1}$ | $\mu_{P1}$ | T-statistic | Degrees of freedom | p-value |
| -1.61 | -1.87 | 20.183 | 249 | $< 2.2 e^{-16}$ |

The spatial aggregation of the intensity of losses and gains per region, as well as their change from the first to the second half of the decade, is displayed in Fig.S28.

**Fig.S29. Average intensity of AGC losses and gains in human-influenced land (top), and changes between the first to the second half of the decade (bottom).** All values are displayed as absolute values.

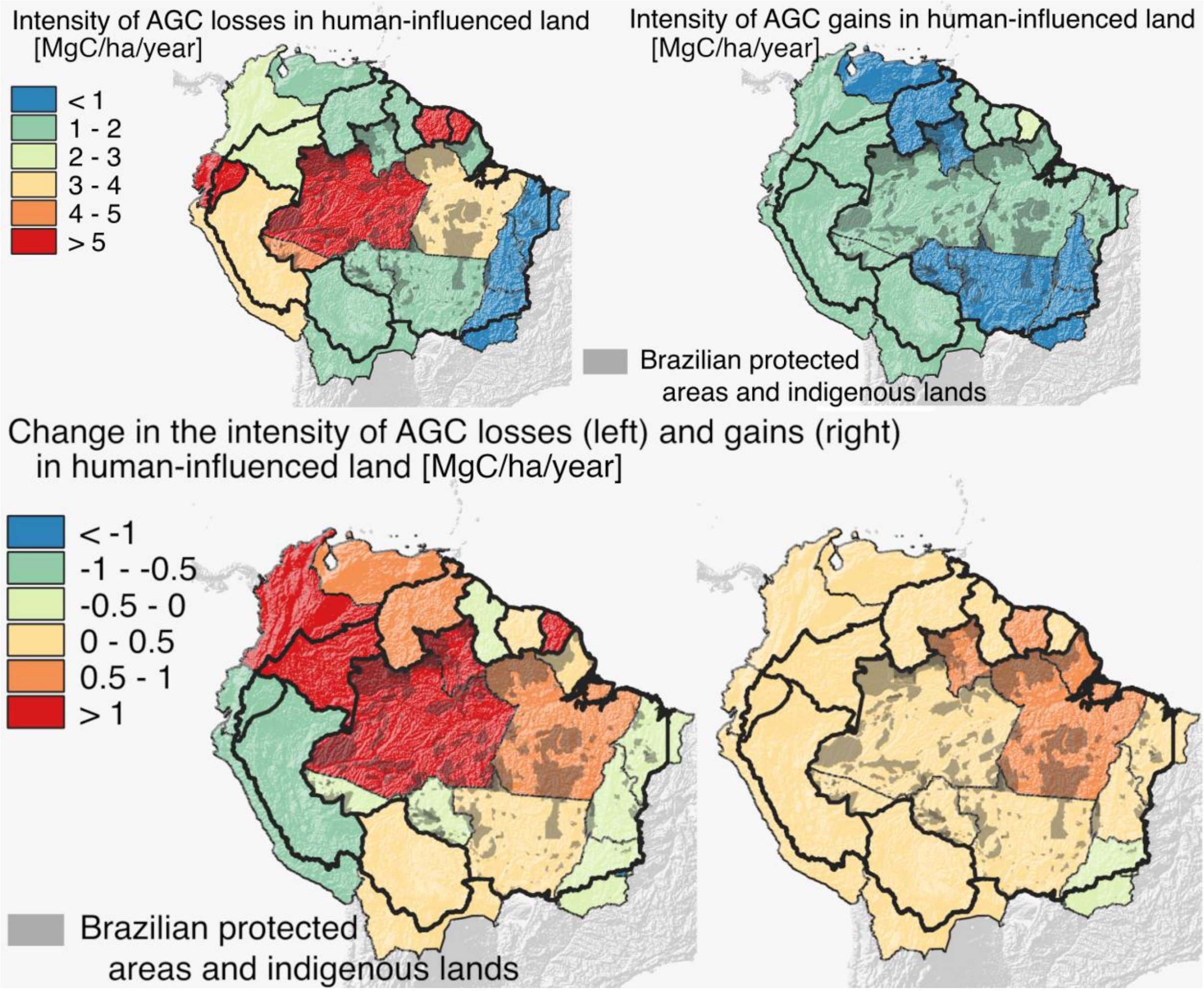

## References

[1] Fendrich, A. N. et al. A scalable method for the estimation of spatial disaggregation models. Computers & Geosciences 166, 105161 (2022).

[2] Wood, S. N. Generalized additive models. Chapman and Hall/CRC eBooks (2017). doi:10.1201/9781315370279.

[3] Stasinopoulos, D. et al. Flexible Regression and Smoothing: Using GAMLSS in R. CRC Press (2017). doi:10.1201/b21973.

[4] Miller, D. et al. Understanding the Stochastic Partial Differential Equation Approach to Smoothing. Journal of Agricultural, Biological and Environmental Statistics 25, 1-16 (2020).

[5] van Buuren, S. & Fredriks, M. Worm plot: a simple diagnostic device for modelling growth reference curves. Statistics in Medicine v.20, i.8, 1259-1277 (2001).

[6] Santoro M. et al. Algorithm Theoretical Basis Document (ATBD, version 4.0, European Space Agency). https://climate.esa.int/media/documents/D2_2_Algorithm_Theoretical_Basis_Document_ATBD_V4.0_20230317.pdf (2023)

[7] Xu, L. et al. Changes in global terrestrial live biomass over the 21st century. Science Advances 7, eabe9829 (2021).

[8] Hubau, W. et al. Asynchronous carbon sink saturation in African and Amazonian tropical forests. Nature, 579, 80-87 (2020).

[9] Brienen, R. et al. Long-term decline of the Amazon carbon sink. Nature 519, 344–348 (2015).

[10] Araza, A. et al. Past decade above-ground biomass change comparisons from four multi-temporal global maps. International Journal of Applied Earth Observation and Geoinformation 118, 103274 (2023).

[11] Araza, A. et al. A comprehensive framework for assessing the accuracy and uncertainty of global above-ground biomass maps. Remote Sensing of Environment 272, 112917 (2022).

[12] Vancutsem, C. et al. Long-term (1990–2019) monitoring of forest cover changes in the humid tropics. Science Advances 7, eabe1603 (2021).

[13] Nunes, S. et al. Unmasking secondary vegetation dynamics in the Brazilian Amazon. Environmental Research Letters 15, 034057 (2020).

[14] Instituto Nacional de Pesquisas Espaciais. PRODES – Amazônia. http://www.obt.inpe.br/OBT/assuntos/programas/amazonia/prodes (2023)

[15] Mašić, A. et al. Shape constrained splines as transparent black-box models for bioprocess modeling. Computers & Chemical Engineering, 99, 96-105 (2017).

[16] Pya, N., & Wood, S. N. Shape constrained additive models. Statistics and computing, 25, 543-559 (2015).

[17] Ometto, J. P. et al. A biomass map of the Brazilian Amazon from multisource remote sensing. Scientific Data, 10(1), 668 (2023).

[18] Asner, G. P. et al. High-resolution forest carbon stocks and emissions in the Amazon. Proceedings of the National Academy of Sciences of the United States of America 107, 16738–16742 (2010).
